# Metabolic Profile and Dynamic Characteristics of Ziziphi Spinosae Semen Flavonoids During Biotransformation and Their Effect on Human Gut Microbiota In Vitro

**DOI:** 10.3390/foods15173077

**Published:** 2026-08-30

**Authors:** Ruiqi Yang, Haolin Zhang, Yadan Mo, Conghui Wang, Jinchun Wu, Lijun Guo, Xiangping Pei, Yan Yan, Chenhui Du

**Affiliations:** 1 School of Chinese Materia Medica, Shanxi University of Chinese Medicine, Taiyuan 030619, China; yangruiqi@sxtcm.edu.cn (R.Y.); 13294592426@163.com (H.Z.); moyadan5533@163.com (Y.M.); w15971113078@163.com (C.W.); wjc19950426@163.com (J.W.); guolijun@sxtcm.edu.cn (L.G.); peix69@163.com (X.P.); 2 Modern Research Center for Traditional Chinese Medicine, Shanxi University, Taiyuan 030006, China

**Keywords:** Ziziphi spinosae semen flavonoids, human gut microbiota, biotransformation, metabolic profile, short-chain fatty acids

## Abstract

The Ziziphi spinosae semen flavonoids (ZSSF) possess significant nutritional value and exhibit a wide range of health benefits, exerting positive effects on diseases related to the central nervous system. Given the excellent bioactivity and limited absorption of ZSSF, elucidating the underlying mechanism of ZSSF is imperative. In this study, in vitro fecal microbial culture fermentation was performed to investigate the metabolism of ZSSF and its effect on the gut microbiota. A total of 45 chemical constituents were identified by Ultra-performance liquid chromatography–quadrupole time-of-flight–tandem mass spectrometry (UPLC-Q-TOF-MS/MS), and possible biotransformation pathways were postulated. The degradation rates of spinosin and 6‴-feruloylspinosin were 2.53 ± 0.07 μg/mL/h and 0.79 ± 0.06 μg/mL/h, respectively, with swertisin accumulating to 55.00 ± 3.55 μg/mL at 24 h before declining. The abundance of *Firmicutes* increased from 8.42% at 0 h to 48.26% in the ZSSF fermentation group at 48 h, while *Actinobacteria* and *Proteobacteria* decreased from 60.02% and 30.25% to 23.46% and 20.02%, respectively. Furthermore, gas chromatography–mass spectrometry (GC-MS) was used to analyze short-chain fatty acids (SCFAs), revealing that ZSSF was associated with higher butyrate concentration at 48 h under the tested batch-fermentation conditions, with significantly higher levels observed in the ZSSF fermentation group compared to the control (1.34 ± 0.03 vs. 1.10 ± 0.05 mmol/L, *p* < 0.05). This study provides the first integrated analysis of ZSSF metabolism, gut microbiota modulation, and SCFAs production in a single in vitro fermentation model, offering a comprehensive understanding of the gut microbiota-mediated biotransformation of ZSSF and its metabolic consequences. Notably, these findings are derived from an in vitro batch fermentation model, and therefore, in vivo validation is required to confirm the physiological relevance of these observations. Collectively, this study provides insight into the exploration of the metabolic functions and mechanisms of ZSSF.

## 1. Introduction

Ziziphi spinosae semen (ZSS) is the dried ripe seed of *Ziziphus jujuba* Mill. var. *spinose* (Bunge) Hu ex H. F. Chou from the *Rhamnaceae* family, which has been regarded as a functional food with a favorable safety profile [1]. One of the main bioactive components of ZSS is flavonoids, which have potent sedative, hypnotic, antidepressive, and anxiolytic properties, as well as improving learning and memory [2]. Currently, more than 67 flavonoids have been identified, and the content of Ziziphi spinosae semen flavonoids (ZSSF) can reach 1.59% in ZSS [3]. ZSSF primarily consist of flavone-*C*-glycosides, such as spinosin and its acylated derivatives like 6‴-feruloylspinosin, as well as flavone-*O*-glycosides like kaempferol-3-*O*-rutinoside. Among these, flavone *C*-glycosides are the major constituents of ZSSFs [4].

Our previous results found that spinosin, 6‴-feruloylspinosin and swertisin were present at the lower limit of quantification in serum [5], while high contents of spinosin (295.22 ± 40.28 ng/mL), 6‴-feruloylspinosin (258.68 ± 109.10 ng/mL), and swertisin (29.24 ± 9.28 ng/mL) were found in the small intestine [6]. Therefore, the paradox between the low bioavailability and high bioactivity of ZSSF has been noted, suggesting that the intestine might be the target organ of ZSSF. However, knowledge regarding the interaction between gut microbiota and ZSSF remains lacking, including the metabolic profile and dynamic characteristics of ZSSF, as well as their effects on gut microbiota metabolism. However, the specific mechanisms by which ZSSF exert their effects in the intestine—particularly the role of gut microbiota in their metabolism—remain poorly understood. Thus, it is necessary and meaningful to study ZSSF in the context of gut microbiota.

The interaction between flavonoids and gut microbiota is reciprocal. In the intestine, including both the small intestine and colon, certain strains of gut microbiota possess enzymes capable of metabolizing flavonoids [7]. Previous studies showed that flavonoids can be converted into various phenolic metabolites with different structures and potential functions by gut microbiota, which play an essential role in human health [8]. The gut microbiota encodes a broad diversity of enzymes, including β-glucosidases, α-rhamnosidases, and C-glycosidases, which are responsible for the deglycosylation, demethylation, dehydroxylation, and C-ring cleavage of flavonoids. These enzymatic reactions convert flavonoid glycosides into absorbable aglycones and further into smaller phenolic acid metabolites via ring fission and dehydroxylation, thereby enhancing their bioavailability and bioactivity [9]. Notably, the gut microbiota plays a particularly critical role in the metabolism of flavonoids that escape absorption in the upper gastrointestinal tract, with an estimated 90–95% of ingested polyphenols reaching the colon, where they undergo extensive microbial transformation [10]. Despite this broad understanding of the general enzymatic mechanisms, the specific metabolic profile and dynamic characteristics of complex mixtures like ZSSF and their precise interplay with the gut microbial ecosystem—particularly the temporal changes in microbial composition and metabolic outputs—remain largely unexplored. For example, flavone-*C*-glycosides in ZSSF (6‴-feruloylspinosin and 6‴-p-coumaroylspinosin) can be transformed into spinosin and swertisin by gut microbiota [11,12]. Short-chain fatty acids (SCFAs), the principal metabolites generated from gut microbiota, have been shown to serve as key mediators in a range of neurological conditions via microbiota–gut–brain communication [13]. Acetic acid, propionic acid and butyric acid have been shown to be involved in altering levels of γ-aminobutyric acid (GABA), glutamate, dopamine and noradrenaline, which play a significant role in physiology and behavior, including insomnia, anxiety and depression [14,15,16]. Thus, comprehending the intrinsic correlation between gut microbiota metabolism and biotransformation of ZSSF may provide deeper insights into their association with human health.

While the pharmacological activities of ZSSF have been extensively documented, the role of gut microbiota in their metabolism remains largely unexplored, particularly regarding the detailed biotransformation pathways, the dynamic changes in microbial community composition, and the resulting impact on SCFAs production. Three key knowledge gaps remain: (1) the complete metabolic pathway of ZSSF mediated by human gut microbiota has not been systematically characterized; (2) the dynamic effects of ZSSF on gut microbial community composition during fermentation are unknown; and (3) the impact of ZSSF on SCFAs production, a key functional output of the microbiota, has not been investigated. In vivo (human or animal) intervention trials are effective in elucidating flavonoid bioactivity. However, they are very time consuming and laborious. Instead, in vitro fermentation is an effective alternative for examining colon behavior of bioactive substances due to its speed, convenience and ethically unrestricted nature [17]. To address these gaps, gut microbiota fermentation in vitro was conducted with the aim to: (1) identify, quantify and assess the released and metabolized polyphenols from ZSSF; (2) propose possible biotransformation pathways of ZSSF mediated by gut microbiota; (3) investigate the changes in fecal microbiota community structure and SCFAs content during in vitro fecal fermentation. We hypothesize that ZSSF will be sequentially degraded by gut microbiota through deglycosylation, ring fission, and dehydroxylation, and that this biotransformation will selectively modulate the gut microbial composition and enhance SCFAs production. Finally, the relationship between SCFAs contents and microbial community structure involved in ZSSF metabolism was investigated, providing insight into the exploration of their metabolic functions and the mechanisms of ZSSF. To our knowledge, this is the first study to comprehensively integrate the metabolic profiling of ZSSF, the dynamic shifts in gut microbial community composition, and the functional output of SCFAs production in a single in vitro human fecal fermentation model. This integrated approach provides a pathway-level understanding of ZSSF biotransformation rather than focusing on individual compounds alone.

## 2. Materials and Methods

### 2.1. Materials and Reagents

Reference standards of swertisin, spinosin, 6‴-feruloylspinosin, rutin, vitexin and quercetin were purchased from Baoji Herbest Bio-Tech Co., Ltd. (Baoji, China). Kaempferol, apigenin, 3-phenylpropionic acid (3-PPA), m-hydroxyphenylpropionic acid (*m*-HPPA) and p-hydroxyphenylpropionic acid (*p*-HPPA) were supplied by Shanghai Yuan Ye Biotechnology Co., Ltd. (Shanghai, China). SCFAs standards (acetic acid, propionic acid, butyric acid, valeric acid, isobutyric acid, isovaleric acid and 2-ethylbutyric acid) were purchased from Tokyo Chemical Industry Co., Ltd. (Tokyo, Japan). All other reagents were of analytical grade. ZSS was provided by Shanxi Zhendong Chinese Herbal Development Co., Ltd. (Changzhi, China). The voucher specimen (No. 20220902) was preserved at Shanxi University of Chinese Medicine.

### 2.2. Preparation of Ziziphi Spinosae Semen Flavonoids

The preparation of ZSSF was performed according to a published method [18]. The dried ZSS were powdered and then degreased with petroleum ether twice, for 1 h each time. The residue was dried and extracted twice with 70% ethanol for 2 h. The ethanol extract was combined and concentrated to a final concentration of 1 g/mL by vacuum rotary evaporation. Then, the concentrated extracts were re-extracted four times with water-saturated n-butanol. After that, the combined n-butanol layers were concentrated and dried to obtain the extract, which was then dissolved in distilled water to achieve a ZSS concentration of 0.5 g/mL. The extract was purified with D-101 macroporous resin and then eluted with 30% ethanol and water. The 30% ethanol fraction was collected and then freeze-dried to obtain ZSSF for further experiments. The total flavonoid content in the ZSSF extract was determined to be 58% using the colorimetric method with spinosin as the reference standard.

### 2.3. Culture Fermentation of Ziziphi Spinosae Semen Flavonoids by Gut Microbiota

The culture fermentation of gut microbiota was carried out according to a previous study [19]. Fresh fecal samples were collected from six healthy adult human volunteers (three men and three women, 25 to 40 years of age) with a body mass index (BMI) within the normal range (18.5–24.9 kg/m^2^), who were non-smokers, omnivorous, and had not used any probiotics or antibiotics in the three months prior to the study. Dietary and lifestyle information was collected using a standardized questionnaire covering habitual diet, physical activity, and medical history. They had no history of gastrointestinal, metabolic, or neurological disorders. The study protocol was approved by the Institutional Review Board of Shanxi University of Chinese Medicine (Approval No. 2023LL326). Written informed consent was obtained from all participants prior to sample collection. The feces were suspended in sterilized DPBS (0.1 M, pH 7.0) to prepare a fecal homogenate (10%, *w*/*v*). A fecal slurry was then prepared from the fecal homogenate by filtering it through clean gauze for the fermentation experiment. Gifu Anaerobic Medium (GAM) broth was dissolved in water and sterilized under high pressure (0.12 MPa) and temperature (115 °C) for 15 min. Before use, sterile 0.1% vitamin K1 and sterile hemin were added to the medium. Then, 9 mL of the fecal slurry was added to a screw tube containing 54 mL of GAM medium and incubated in an anaerobic incubator at 37 °C for 9 h to revitalize the microbiota. Subsequently, 1.4 mL of ZSSF solution (ZSSF dissolved in 30% ethanol at a final concentration of 17.0 mg/mL) was added to the above system as the ZSSF fermentation group (the concentration of 17.0 mg/mL ZSSF was selected based on our previous optimization studies, in which this concentration yielded detectable metabolite formation while maintaining physiologically relevant conditions for in vitro fermentation [20]). A control group without ZSSF solution was also prepared (CON). In addition, another control group consisted of GAM medium and ZSSF solution without human gut microbiota. The mixture was incubated in an anaerobic incubator at 37 °C. The incubation experiment was conducted in triplicate as technical replicates using aliquots from the same pooled fecal slurry from the six donors. Samples were collected at 0, 2, 6, 12, 24, and 48 h and were stored at −80 °C for further analysis.

### 2.4. Determination of Flavonoids and Their Metabolites

Qualitative and quantitative analyses were performed using a Shimadzu Prominence ultra-performance liquid chromatography (UPLC) system （Shimadzu Corporation, Kyoto, Japan）equipped with an ACQUITY UPLC ^®^ HSS T3 column (2.1 × 100 mm, 1.8 μm, Waters Corporation, Milford, MA, USA). The column temperature was set at 40 °C. The mobile phase consisted of phase A (0.1% formic acid) and phase B (acetonitrile) at a flow rate of 0.3 mL/min, with an injection volume of 2 μL. The gradient elution program for phase B was as follows: 0–8 min, 5–17% B; 8–10 min, 17% B; 10–11 min, 17–18% B; 11–12 min, 18–20% B; 12–17 min, 20–23% B; 17–22 min, 23–33% B; 22–25 min, 33–100% B.

Mass spectrometry (MS) analysis was performed using an AB SCIEX 5600^+^ Triple TOF mass spectrometer (Framingham, MA, USA) operated in negative ionization mode with an electrospray ionization (ESI) source. Data acquisition was performed using a combination of full-scan TOF-MS and IDA mode. The optimized conditions were as follows: ionspray voltage, −4.5 kV; declustering potential, −50 V; collision energy, −10 eV; turbo spray temperature, 450 °C; nebulizer gas, 50 psi; heater gas, 50 psi; curtain gas, 30 psi. For IDA, MS/MS acquisition was performed in the mass range of *m*/*z* 50–1500 Da. A collision energy of −35 eV with a ±15 eV spread was used for MS/MS fragmentation [6]. The four highest intensity precursors (i.e., ion intensity > 100 cps) were monitored in each cycle, and dynamic background subtraction was enabled. Analyst TF 1.8.1 software was used for data acquisition, and SCIEX OS 1.5 was used for data processing (both from SCIEX, Framingham, MA, USA). Qualitative analysis was conducted by comparing the results with online databases and corresponding standards.

### 2.5. Determination of Short-Chain Fatty Acids Content

Briefly, 1 mL of 0.5% sulfuric acid was added to each sample to adjust the pH to 2–3. Subsequently, 3 mL of a 1:1 methanol–water solution was added, and the mixture was vortexed for 3 min, then centrifuged at 13,000 rpm (16,200× *g*) for 20 min. Then, 2 mL of the supernatant was aspirated. Next, 10 μL of a 2-ethylbutyric acid internal standard solution was added to the supernatant, and the mixture was filtered through a 0.45 μm microporous membrane for subsequent analysis.

Short-chain fatty acids were quantified using a previously reported GC-MS method [20] (Agilent 7890 B GC-MS system coupled with an Agilent 5977 mass selective detector (Agilent, Santa Clara, CA, USA) and equipped with a DB-FFAP capillary column (30 m × 0.25 mm × 0.25 μm)). The temperatures of the injector, ion source, quadrupole, and GC-MS interface were set at 175, 230, 150, and 220 °C, respectively. The flow rate of the helium carrier gas was 1 mL/min. A 0.5 μL sample was injected with a solvent delay time of 3 min and a split ratio of 20:1. The initial column temperature was 90 °C, held for 1 min, then ramped to 200 °C at a rate of 10 °C/min and held for 13 min. Ionization was carried out in electron impact (EI) mode at 70 eV. MS data were acquired in full-scan mode over the mass range of *m*/*z* 50–500.

### 2.6. Analysis of Gut Microbiota

The microbial community compositions of samples from three groups (CON 0 h, CON 48 h, and ZSSF fermentation group 48 h) with three replicates per group were investigated during in vitro fermentation using 16S rRNA gene sequencing and bioinformatic analysis. The 0 h time point was selected to represent the baseline microbial community before fermentation, while the 48 h time point was chosen as the end point to capture the maximum effects of fermentation on community structure based on the preliminary time-course results. The samples prepared above were sent to Shanghai Personal Biotechnology Co., Ltd. (Shanghai, China) for analysis of microbiota composition. High-throughput sequencing was performed using the Illumina MiSeq platform. The V3–V4 region of the 16S rRNA was selected for amplification. The clean reads were clustered to produce Amplicon Sequence Variants (ASVs) using the QIIME2 platform. In addition, the sequenced reads and ASVs formed the basis of all results. Alpha diversity reflects the species richness and evenness within individual samples. For beta diversity, principal coordinate analysis (PCoA) and non-metric multidimensional scaling (NMDS) were performed to compare microbial the community composition across different sample groups. The Binary Jaccard and Bray–Curtis dissimilarity metrics were calculated using QIIME 2 (version 2024.10), and the resulting distance matrices were subjected to ordination analyses. The PCoA and NMDS results were visualized as two-dimensional scatter plots to illustrate the dissimilarities among groups.

### 2.7. Statistical Analysis

The results were expressed as the mean ± standard deviation (SD). The illustrations were created using GraphPad Prism version 8.0 (GraphPad Software, San Diego, CA, USA). Due to the destructive sampling design of the in vitro fermentation experiment, where samples at each time point were collected from separate fermentation vessels rather than from the same biological replicate repeatedly over time, a repeated-measures model was not considered adopted. Therefore, data were compared by one-way analysis of variance (ANOVA) with time as a fixed factor, followed by a post hoc Duncan’s test for multiple comparisons, using SPSS 19.0 (IBM, New York, USA). Spearman’s correlation analysis was performed using the OmicStudio tools available at https://www.omicstudio.cn/tool (accessed on 15 March 2024. *p* < 0.05 was regarded as statistically significant). Before the analysis of variance, we tested the normal distribution and homogeneity of variance of the data. *p* > 0.05 for the data obeyed the test of normal distribution and homogeneity of variance.

## 3. Results and Discussion

### 3.1. Chemical Characterization of Compounds and the Metabolites in Ziziphi Spinosae Semen Flavonoids

**Figure 1:**
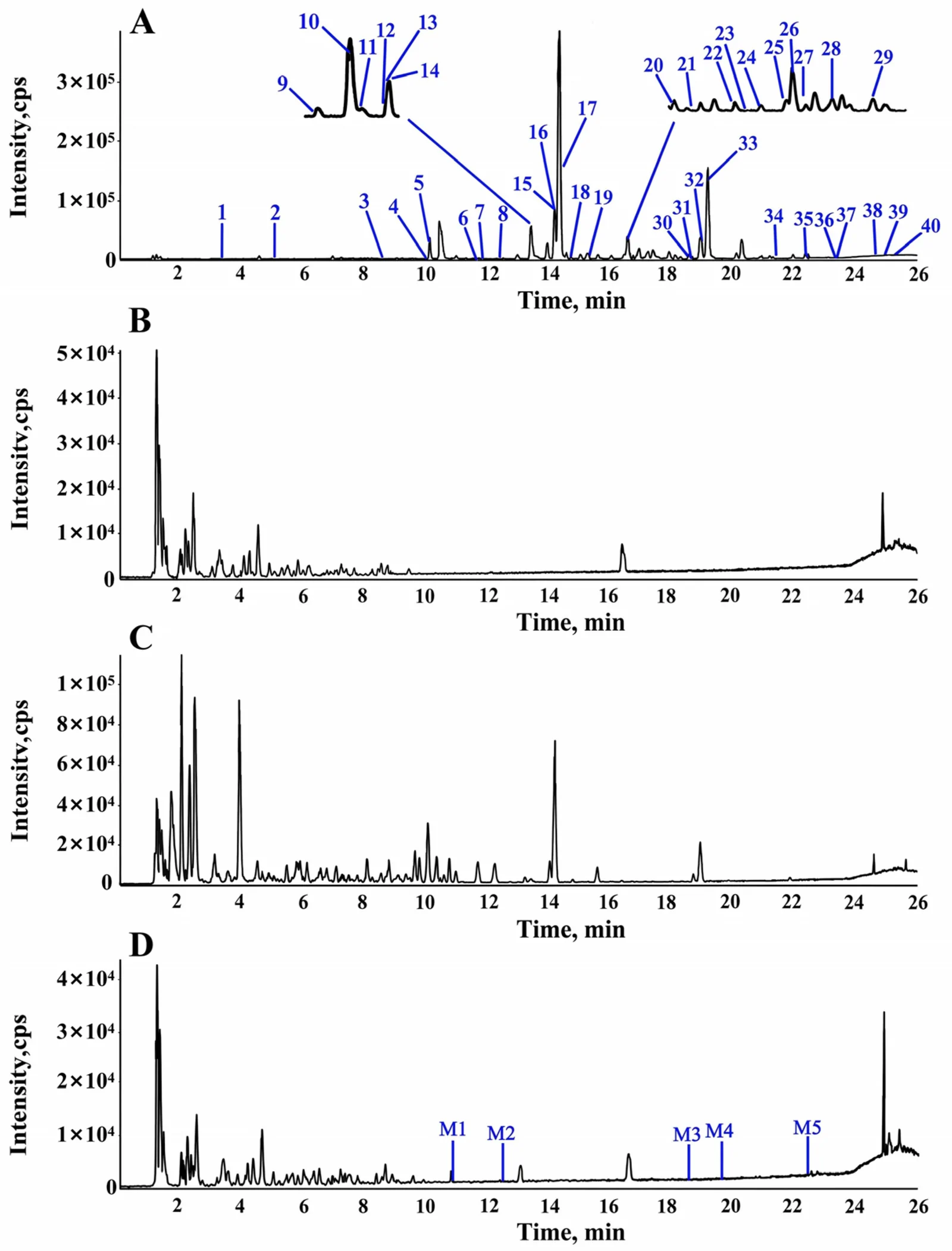
Base peak chromatograms (BPCs) of different groups: (**A**) ZSSF; (**B**) control group without ZSSF solution (CON); (**C**) group without human gut microbiota; and (**D**) ZSSF group incubated for 48 h (ZSSF fermentation group). (Numbers in the figure indicate peak numbers corresponding to those listed in Table S1).

A total of 45 chemical constituents were identified or tentatively characterized in ZSSF by UPLC-Q-TOF-MS/MS equipped with an electrospray ionization source operating in negative ionization mode. Among these, 31 flavonoids, nine phenolic acids, and five other compounds were identified in ZSSF and their metabolites. The details are listed in Table S1. The chemical structures of ZSSF and their metabolites are shown in Figure S1, and the base peak chromatograms are presented in Figure 1.

#### 3.1.1. Flavonoid C-Glycosides

**Figure 2:**
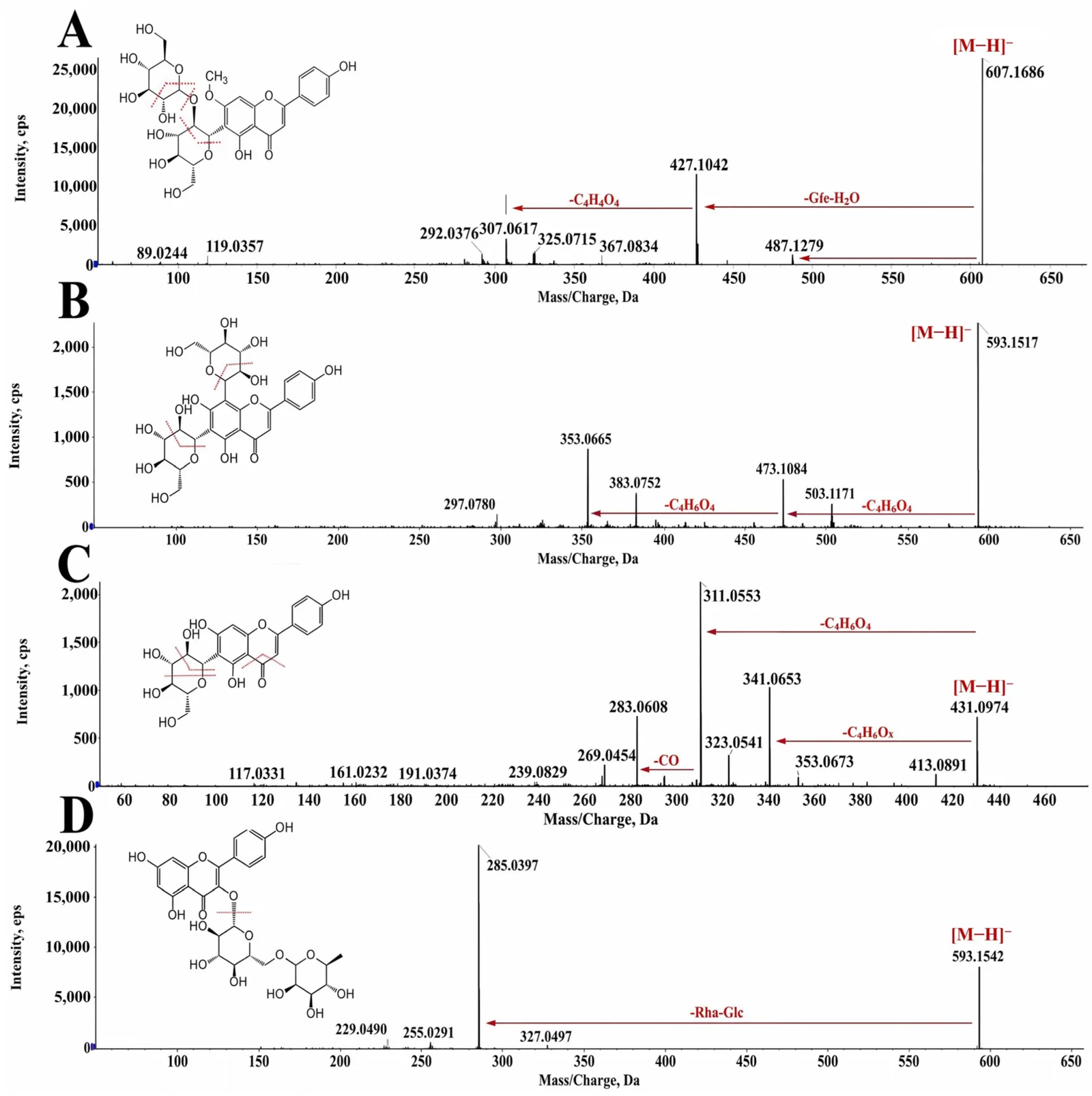
MS/MS spectra of ZSSF with their proposed fragmentation pathways in negative ion mode. (**A**) Spinosin; (**B**) Vicenin II; (**C**) Isovitexin; (**D**) Kaempferol-3-O-rutinoside.

According to the aglycon skeleton and the position of the glycosidic linkage, ZSSF were categorized into two groups: flavonoid *C*-glycosides and flavonoid *O*-glycosides. Furthermore, the flavonoid *C*-glycosides from ZSS were further subdivided to genkwanin *C*-glycosides and apigenin *C*-glycosides. For the genkwanin *C*-glucosides, spinosin (**17**), swertisin (**20**), and 6‴-feruloylspinosin (**33**) were identified by comparison with the retention times of the corresponding reference standards. Compound **33** exhibited a fragment ion at *m*/*z* 607.1719, which was generated by the loss of a feruloyl group from the ion at *m*/*z* 783.2146. It was also fragmented into an ion at *m*/*z* 427.1040 by the loss of a feruloyl group and a glucose unit; therefore, it was identified as 6‴-feruloylspinosin. Spinosin (**17**), a typical genkwanin 6-*C*-glycoside, showed a [M − H]^−^ ion at *m*/*z* 607.1686, which fragmented into three characteristic ions at *m*/*z* 427.1042, *m*/*z* 307.0617, and *m*/*z* 292.0376 (Figure 2A). The fragment ion at *m*/*z* 427.1042 corresponded to [M − H-C_6_H_10_O_5_-H_2_O]^−^, derived from the ion at *m*/*z* 607.1686 by the loss of a glucosyl unit and one molecule of H_2_O (180 Da) [21]. The fragment ion at *m*/*z* 307.0617, originating from the ion at *m*/*z* 427.1042, was observed following the loss of C_4_H_8_O_4_ (120 Da); subsequent loss of CH_2_ yielded a typical product ion at *m*/*z* 292.0376 [22]. Moreover, the precursor ion of compound **15** was at *m*/*z* 607.1660, indicating that it was an isomer of spinosin, and it was therefore reasonably assigned as isospinosin (a genkwanin *C*-8-glycoside) [23].

Regardless of whether a sugar moiety was attached to the acyl group, the acyl derivatives of spinosin were divided into two parts. Compounds **9**, **11**, **13**, **14**, and **21** initially lost a glucose unit (162 Da) or a rhamnose unit (146 Da) in negative ion mode and subsequently exhibited mass spectral behavior similar to that of 6‴-feruloylspinosin. Likewise, compounds **23**, **26**, **30**, **31**, **32**, **34**, **35**, and **37** first underwent the loss of an acyl group, such as a phaseoyl group (262 Da) or a sinapoyl group (206 Da), and then generated common fragment ions at *m*/*z* 607, 427, and 307, which exhibited mass spectral behavior similar to that of 6‴-feruloylspinosin [3].

Regarding apigenin-*C*-glucosides, compound **4** (*m*/*z* 593.1505) was identified as vicenin II (apigenin 6,8-di-*C*-glucoside) by comparing with the standard. The ion peaks of vicenin II (**4**) at *m*/*z* 473.1084 [M – H − 120]^−^ and *m*/*z* 353.0665 [M – H – 120 − 120]^−^ were generated due to the breakage of one or two glucose molecules (Figure 2B) [24]. Losses of 90 Da (C_3_H_6_O_3_) and 120 Da (C_4_H_8_O_4_) are characteristic fragment ions of *C*-glycosides. In negative ion mode, vitexin (**16**) (apigenin 8-*C*-glucoside, t_R_ = 14.30 min, *m*/*z* 431.0980) and isovitexin (**19**) (apigenin 6-*C*-glucoside, t_R_ = 14.57 min, *m*/*z* 431.0974, Figure 2C) are a pair of isomers. The ions at *m*/*z* 341.0653 and 311.0553 were observed as characteristic ions in the MS/MS mode, which were produced by the loss of C_3_H_6_O_3_ ([M – H − 90]^−^) and C_4_H_8_O_4_ ([M – H − 120]^−^) from the ion at *m*/*z* 431 [25]. Compounds **10**, **27**, and **28** were tentatively characterized by comparison with available references as isovitexin-2′′-*O*-*β*-*D*-glucopyranoside, isovitexin-2′′-*O*-(6-*p*-coumaroyl)-glucopyranoside, and isovitexin-2′′-*O*-(6-feruloyl)-glucopyranoside, respectively [3].

#### 3.1.2. Flavonoid O-Glycosides

Meanwhile, five compounds (**12**, **18**, **24**, **36**, and **39**) were identified as flavonoid *O*-glycosides. Kaempferol-3-*O*-rutinoside (**24**) exhibited a [M−H]^−^ ion at *m*/*z* 593.1542 and produced *m*/*z* 285.0397 [M – H – Rha − Glu]^−^ as the high-intensity peak (Figure 2D). Similarly, compound **39** was identified as kaempferol, with a [M−H]^-^ ion at *m*/*z* 285.0398, which yielded a fragment ion at *m*/*z* 223.0297 resulting from the loss of H_2_O followed by the loss of CO_2_ [26]. Based on comparison with the literature, compound **12** was characterized as camelliaside B [27].

#### 3.1.3. Phenolic Acids and Other Compounds

There were six phenolic compounds (**1**, **2**, **3**, **6**, **7**, and **8**) identified in ZSSF. Compounds **1**, **2**, and **6** were assigned as gallic acid, protocatechuic acid, and *p*-coumaric acid, respectively. They exhibited product ions at *m*/*z* 125.0222, *m*/*z* 108.0216, and *m*/*z* 119.0507, respectively, which correspond to the neutral loss of CO_2_ (44 Da) from the parent ions [28]. Catechin (**3**) produced a deprotonated ion [M−H]^-^ at *m*/*z* 289.0713 and lost a CH_2_CHOH group (*m*/*z* 245.0807) [29]. Ethyl gallate (**7**) showed a [M−H]^-^ ion at *m*/*z* 197.0455 and fragment ions at *m*/*z* 169.0122 ([M – H − 28]^−^) and *m*/*z* 125.0246 ([M – H – 28 − 44]^-^), corresponding to the loss of an ethyl unit and a gallic acid unit, followed by the loss of a CO_2_ group [30]. The precursor ion of vanillic acid (**8**) was observed at *m*/*z* 167.0353, and it exhibited product ions at *m*/*z* 152.0135 and *m*/*z* 108.0218, corresponding to the neutral loss of CH_3_ (15 Da) or of CH_3_ and CO_2_ (59 Da) from the parent ion [31].

#### 3.1.4. Tentative Identification of Microbial Metabolites

The potential metabolites were identified through comparative analysis of ZSSF, CON, and the group without human gut microbiota (Figure 1). A total of three phenolic acid metabolites, namely, *p*-HPPA (**M1**) (*m*/*z* 165.0536), *m*-HPPA (**M2**) (*m*/*z* 165.0533), and 3-PPA (**M5**) (*m*/*z* 149.0572), were identified in the ZSSF fermentation group by comparison with reference standards. Moreover, the compounds 6‴-feruloylspinosin-CH_2_O (M3) and swertisin +4H (M4) were not detected in ZSSF over the entire 48 h of the fermentation period and were therefore tentatively identified as potential metabolites. However, their assignment is based solely on the mass shift (loss of CH_2_O for M3 and gain of 4H for M4) relative to their parent compounds, and their structures were not confirmed owing to the lack of authentic standards. Consequently, these identifications should be considered provisional (Figure 1D).

### 3.2. Analysis of the Contents and Metabolic Pathways of Ziziphi Spinosae Semen Flavonoids

**Figure 3:**
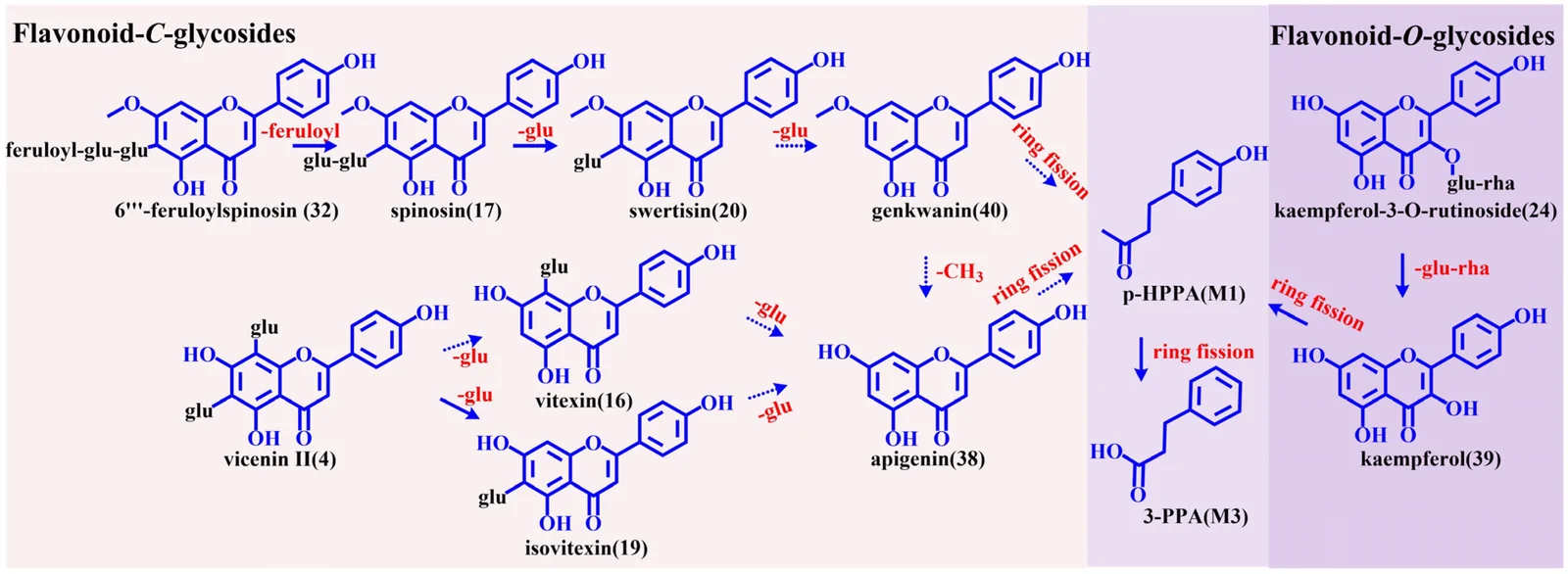
Potential metabolic pathways of ZSSF mediated by human gut microbiota (Solid arrows represent confirmed pathways, and dashed arrows represent speculated pathways).

The proposed metabolic pathways of ZSSF are displayed in Figure S2 based on the results of the above experiment, including the flavonoid-*C*-glycoside pathway and the flavonoid-*O*-glycoside pathway. To further elucidate the metabolism of ZSSF, 10 flavones were selected for quantitative analysis. These included the major parent compounds representative of the different structural classes found in ZSSF: vicenin II (**4**), vitexin (**16**), spinosin (**17**), isovitexin (**19**), swertisin (**20**), kaempferol-3-O-rutinoside (**24**), 6‴-feruloylspinosin (**33**), Apigenin (**38**), kaempferol (**39**), and Genkwanin (**40**). In addition, the three previously identified key metabolites, *p*-HPPA (**M1**), *m*-HPPA (**M2**), and 3-PPA (**M5**), were also analyzed to monitor the downstream degradation process. The proposed metabolic pathways of ZSSF are shown in Figure 3.

All validation parameters obtained for the analytical method were in agreement with the acceptance criteria. The correlation coefficients (*r*) of the analytical curves for all 13 flavones and metabolites were higher than 0.9990 (Table S2). The intra-day precision showed relative standard deviations (RSDs) ranging between 0.47% and 5.70%, and the inter-day precision ranged from 1.43% to 6.74% (Table S3). For spinosin derivatives and isovitexin derivatives without reference standards, their concentrations were evaluated as relative abundances in vitro using the peak area ratio of the metabolites to the internal standard. The concentrations of camelliaside B (**12**), isospinosin (**15**), and isoswertisin (**22**) were determined based on the standard curves of kaempferol-3-*O*-rutinoside (**24**), spinosin (**17**), and swertisin (**20**), respectively. Spinosin derivatives (**23**, **30**, **31**, and **32**) were quantified using the standard curve of 6‴-feruloylspinosin (**33**). Isovitexin derivatives (**10**, **27**, and **28**) were quantified using the standard curve of isovitexin (**19**). The changes in their contents during the incubation process are shown in Figure S3 and Table S5.

#### 3.2.1. Metabolic Pathway of Genkwanin C-Glycosides

**Figure 4:**
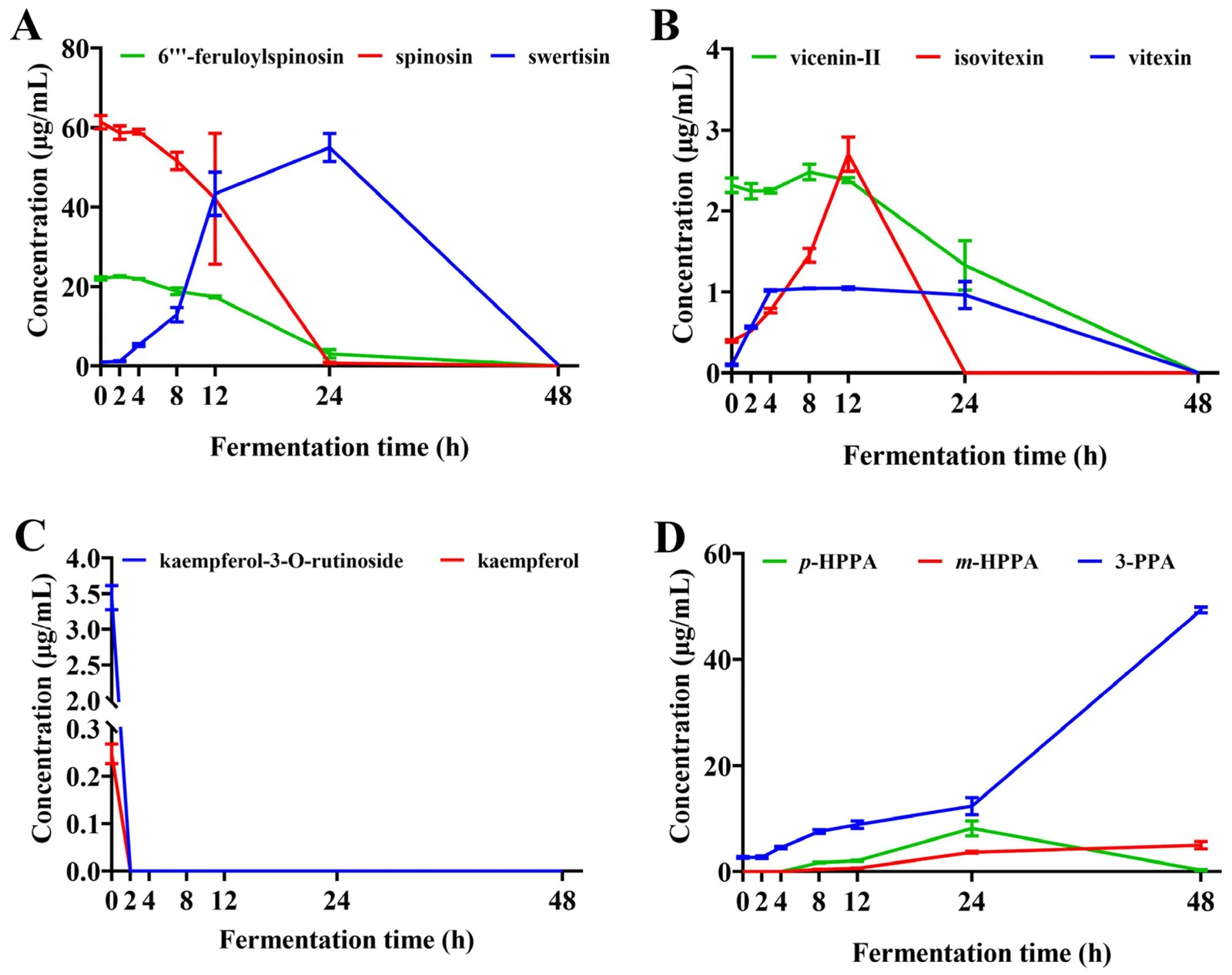
Degradation curves of ZSSF and production curves of metabolites after incubation with human gut microbiota. (**A**) Genkwanin C-glycosides; (**B**) Apigenin C-glycosides; (**C**) Kaempferol O-glycosides; (**D**) Phenolic acids.

As illustrated in Figure 3, 6‴-feruloylspinosin (**33**) underwent degrading to spinosin (**17**) via the cleavage of the feruloyl moiety and was subsequently converted to swertisin (**20**) through the cleavage of the glucose moiety [12]. The degradation curves of the above compounds are shown in Figure 4A (nonlinear fit across 0–48 h). The concentrations of spinosin (**17**) and 6‴-feruloylspinosin (**33**) were measured as 61.38 ± 1.67 μg/mL and 22.00 ± 0.43 μg/mL at 0 h, respectively, and had decreased below the limit of quantification and limit of detection at 48 h (Table S4).

As shown in Figure S2, the derivatives of spinosin (**23**, **30**, **31**, **32**, **34**, and **37**) were degraded to spinosin via ester cleavage, and their concentrations exhibited the same tendency as that of 6‴-feruloylspinosin and decreased during the fermentation process (Figure S3A). Based on these results, the degradation rates of spinosin (**17**) and 6‴-feruloylspinosin (**33**), calculated over the 0–24 h interval, were 2.53 ± 0.07 μg/mL/h and 0.79 ± 0.06 μg/mL/h, respectively, indicating that the hydrolysis rate of glycosidic bonds was faster than that of ester bonds. In contrast, the concentration of swertisin (**20**) continuously increased within 24 h and reached its maximum value (55.00 ± 3.55 μg/mL) at 24 h, after which it was further metabolized to genkwanin, with the concentration decreasing to 0.27 ± 0.01 μg/mL at 48 h (Table S4).

Similarly, as shown in Figure S2, isospinosin (**15**) was transformed into isoswertisin (**22**), which was subsequently metabolized to apigenin (**38**). As displayed in Figure S3B and Table S5, the concentration of isospinosin (**15**) was 7.70 ± 0.50 μg/mL at 0 h, and it was fully metabolized by 4 h. However, isoswertisin (**22**, 8-*C*-glycoside) reached its maximum concentration at 24 h (9.59 ± 0.73 μg/mL), after which the concentration declined from 24 to 48 h. A comparison of the degradation rates of swertisin (**20**) (2.28 ± 0.15 μg/mL/h) and isoswertisin (**22**) (0.40 ± 0.03 μg/mL/h), calculated over the 24–48 h interval during which both compounds were in the degradation phase, indicated that the genkwanin 6-*C*-glycoside was metabolized faster than the genkwanin 8-*C*-glycoside.

#### 3.2.2. Metabolic Pathway of Apigenin C-Glycosides

Likewise, the degradation curves of apigenin *C*-glycosides are shown in Figure 4B. Vicenin II (**4**), vitexin (**16**), and isovitexin (**19**) were all ultimately degraded to apigenin (**38**) [32,33]. As displayed in Table S4, the concentration of vicenin II (**4**) was 2.32 ± 0.09 μg/mL at 0 h and gradually decreased until it fell below the limit of detection at 48 h. Vicenin II (**4**) underwent hydrolyzing to vitexin (**16**) and isovitexin (**19**).

In turn, the concentration of vitexin (**16**) continued to rise within 4 h. Subsequently, vitexin (**16**) entered a stable period from 4 to 24 h, during which its concentration remained essentially unchanged at approximately 1.02 ± 0.01 μg/mL, and then decreased to below the limit of detection at 48 h.

In contrast, the concentration of isovitexin (**19**) increased and reached its maximum at 12 h (2.70 ± 0.21 μg/mL), which may be attributed to the fact that isovitexin derivatives (**10**, **27**, and **28**) can be degraded to isovitexin (**19**) (Figure S3C and Table S5). It subsequently decreased to below the limit of detection at 24 h. In conclusion, the metabolic rate of isovitexin (**19**) (0.23 ± 0.02 μg/mL/h) was faster than that of vitexin (**16**) (0.03 ± 0.00 μg/mL/h), calculated over the 12–24 h interval for isovitexin and the 12–48 h interval for vitexin during their degradation phases, indicating that the apigenin 6-*C*-glycoside was metabolized faster than the apigenin *8*-C-glycoside by the gut microbiota.

#### 3.2.3. Metabolic Pathway of Kaempferol O-Glycosides

Moreover, kaempferol-3-*O*-rutinoside (**24**) and kaempferol (**39**) were only detected at 0 h, with concentrations of 3.44 ± 0.17 μg/mL and 0.25 ± 0.02 μg/mL, respectively (Table S4), and were degraded completely within 2 h (Figure 4C). As shown in Figure S3D and Table S5, the concentration of camelliaside B decreased during the fermentation process, which might be attributed to its degradation to kaempferol-3-*O*-rutinoside via the cleavage of the xylose unit (Figure S2). The metabolic rates of kaempferol-3-*O*-rutinoside and kaempferol were 1.72 ± 0.08 μg/mL/h and 0.12 ± 0.01 μg/mL/h, respectively, calculated over the 0–2 h interval during which both compounds were rapidly degraded to below the limit of detection (Figure 4C).

#### 3.2.4. Formation of Phenolic Acid Metabolites

As shown in Figure 3, we hypothesize that apigenin (**38**), kaempferol (**39**), and genkwanin (**40**) were hydrolyzed to *p*-HPPA (**M1**) via cleavage of the *C*-ring and were then degraded to 3-PPA (**M5**) through dehydroxylation.

In this study, as shown in Table S4, *p*-HPPA (**M1**) reached its maximum concentration (8.19 ± 1.43 μg/mL) at 24 h and then declined gradually, while 3-PPA (**M5**) accumulated over time and ultimately reached 49.32 ± 0.54 μg/mL at 48 h. Furthermore, *m*-HPPA (**M2**) was generated from rutin through deglycosylation, reduction, and ring fission (Figure S2) [34]. As shown in Table S4, the concentration of *m*-HPPA (**M2**) accumulated over 48 h (Figure 4D) and reached 5.02 ± 0.70 μg/mL at 48 h.

These results suggest that *m*-HPPA (**M2**) and 3-PPA (**M5**) were the major end products detected after 48 h of fermentation. However, it is important to note that these compounds may represent the most persistent metabolites within the timeframe of this experiment, but they could potentially undergo further degradation under longer-term incubations or in the presence of a different microbial community composition.

In summary, the gut microbiota-mediated metabolism of ZSSF involved ester cleavage, deglycosylation, demethylation, ring fission, and dehydroxylation. This study reveals a structure-dependent metabolic preference: 6-*C*-glycosides were metabolized faster than their 8-*C*-glycosidic counterparts, while *O*-glycosides (kaempferol-3-*O*-rutinoside) were degraded more rapidly than *C*-glycosides, consistent with the general understanding that *O*-glycosidic bonds are more readily hydrolyzed by gut microbial enzymes compared to *C*-glycosidic bonds [7,35]. The stepwise degradation from flavonoid glycosides to aglycones and ultimately to phenolic acids such as 3-PPA and *m*-HPPA aligns with established colonic metabolic pathways of dietary flavonoids, in which C-ring fission and dehydroxylation generate low-molecular-weight metabolites with enhanced bioavailability [8,36].

### 3.3. Diversity Analysis of Gut Microbiota

#### 3.3.1. Alpha Diversity

Alpha diversity analysis was conducted to evaluate the species richness and diversity of individual samples. As shown in Figure S4A,B, the rarefaction curves of observed species and the Shannon curves tended to flatten out, indicating that the sequencing depth for each sample in this study was sufficient and that the data were therefore robust enough for further analyses.

The Simpson and Shannon indices are frequently used to assess microbial diversity, while the Chao1 and observed species indices are commonly used to reflect species richness. As shown in Figure S4C, when compared with the CON group at 0 h, the Simpson, Shannon, Chao1, and observed species indices of both the CON and ZSSF fermentation groups at 48 h increased significantly over the entire fermentation process from 0 h to 48 h (*p* < 0.05) and reached their maximum values at 48 h. This increase in both groups suggests that the in vitro fermentation process itself promoted microbial community enrichment, likely due to the nutrient-rich GAM medium supporting the growth of diverse bacterial taxa [37]. However, no significant differences were observed between the CON and ZSSF fermentation groups at 48 h. The lack of significant differences between the two groups indicates that ZSSF supplementation did not markedly affect overall species richness or evenness under the current experimental conditions. This is in line with a previous study reporting that certain flavonoid treatments did not significantly alter the overall diversity of fecal microbiota [38].

Nonetheless, it should be noted that alpha diversity measures only community richness and evenness at the species level, and may not capture shifts in specific taxa or functional profiles. Therefore, beta diversity and taxonomic composition analyses were further performed to explore the structural differences induced by ZSSF.

#### 3.3.2. Venn Diagram Analysis

The Venn diagram shows the shared and unique ASVs among the three groups. A total of 527 shared ASVs were detected in all samples (Figure S4D). In addition, 944, 1356, and 1178 unique ASVs were detected in the CON 0 h, CON 48 h, and ZSSF fermentation 48 h groups, respectively. The number of mutual ASVs (1034) between the CON and ZSSF fermentation groups at 48 h was the highest among the three experimental groups.

The marked increase in unique ASVs after 48 h fermentation in both group indicates that the in vitro fermentation process markedly enriched the microbial community, consistent with the alpha diversity results. The relatively high number of shared ASVs between CON 48 h and ZSSF fermentation 48 h suggests that the two groups shared a substantial core microbiota after 48 h of fermentation, which is consistent with the lack of significant differences in alpha diversity between these groups. However, the ZSSF fermentation group also possessed a considerable number of unique ASVs, indicating that ZSSF supplementation selectively enriched certain taxa that were not abundant in the CON group. The presence of 527 ASVs common to all three groups likely represents the core microbiota that persisted throughout the fermentation process regardless of treatment, which may include bacterial species capable of utilizing the basal GAM medium components. These observations suggest that while ZSSF did not drastically alter the overall microbial community structure, it may have selectively modulated specific taxa, warranting further investigation at the taxonomic composition level.

#### 3.3.3. Beta Diversity Analysis

**Figure 5:**
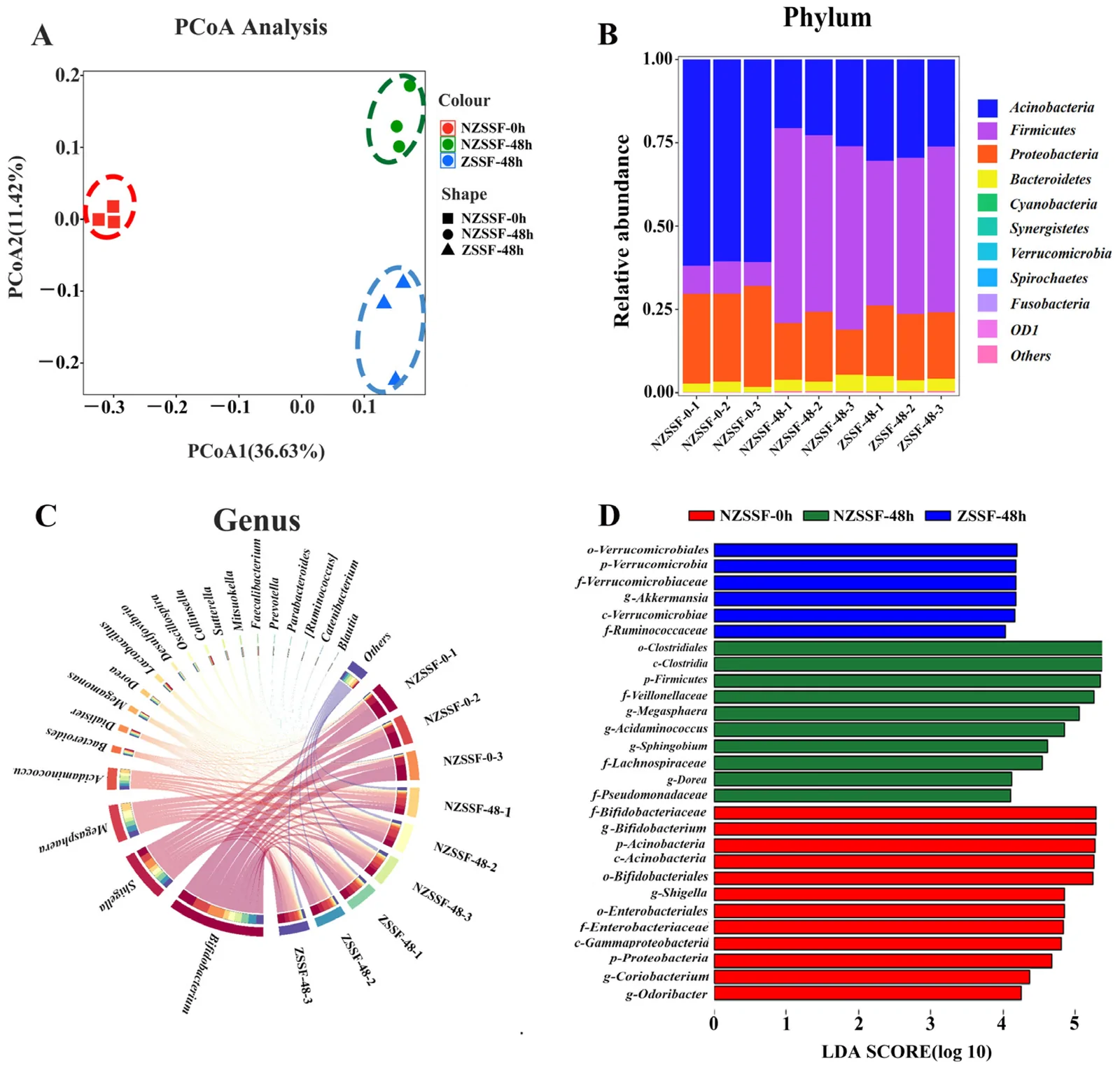
Comparisons of microbiota composition during in vitro colonic fermentation at different time points. (**A**) Principal coordinates analysis (PCoA); (**B**) Relative abundance of the gut microbiota community at the phylum level; (**C**) Gut microbiota taxonomic profiles at the genus level; (**D**) Linear discriminant analysis (LDA) scores for differentially abundant taxa.

Beta diversity analysis was performed to assess dissimilarities in microbial community composition across different samples. PCoA based on the Binary–Jaccard algorithm and NMDS based on the Bray–Curtis algorithm were applied to analyze the beta diversity of the samples (Figure 5A). In Figure 5A, the results demonstrated that the three groups were separated along the PCoA1 and PCoA2 axes in the PCoA model. The two principal axes explained 48.05% of the total variation (PCoA1: 36.63%; PCoA2: 11.42%). The clear separation between 0 h and 48 h groups along PCoA1 (36.63% variance) indicates that fermentation time was the dominant factor shaping microbial community structure, consistent with the alpha diversity changes observed over time. In particular, the samples from the 0 h group and the 48 h groups were significantly separated along the PCoA1 axis. Both the CON 48 h and ZSSF fermentation 48 h samples formed two well-separated clusters distributed along the PCoA2 axis, with clear variability detected among all three groups. The distinct clustering of CON 48 h and ZSSF fermentation 48 h along PCoA2 (11.42% variance) demonstrates that ZSSF supplementation significantly altered the overall microbial community composition beyond the temporal effect. The results obtained from the PCoA analysis were also supported by the NMDS analysis.

Hierarchical clustering trees based on weighted-UniFrac distance were constructed for hierarchical clustering of the samples (Figure S5B). The weighted-UniFrac clustering analysis showed that at a genetic distance of 0.15, the 0 h group and the 48 h groups could be clearly clustered into two classes. However, at a genetic distance of 0.22, the CON 0 h, CON 48 h, and ZSSF fermentation 48 h groups each displayed clear clustering. The hierarchical clustering trees yielded comparable results to those obtained from the PCoA and NMDS analyses. The higher number of unique ASVs in the ZSSF fermentation group and the clear separation in beta diversity collectively suggest that ZSSF selectively modulated specific taxa, even though overall species richness was not significantly different between the two 48 h groups. This underscores the importance of beta diversity analysis in detecting structural changes that alpha diversity alone cannot capture.

#### 3.3.4. Species Composition Analysis

Based on the ASVs obtained from sample sequencing, the gut microbiota compositions of the CON 0 h, CON 48 h, and ZSSF fermentation 48 h groups were plotted as histograms at the phylum level (Figure 5B) and the genus level (Figure 5C). The differences in species structure of the gut microbiota among the groups were compared. As shown in Figure 5B, after 48 h of culture, the structure of the gut microbiota community changed significantly. In agreement with previous studies [39], *Firmicutes*, *Proteobacteria*, and *Actinobacteria* accounted for approximately 99% of the total gut microbiota in the human colonic flora.

The abundance of *Firmicutes* in the CON 48 h group (56.25%) and the ZSSF fermentation 48 h group (48.26%) was significantly increased compared with that in the CON 0 h group (8.42%). Compared with the CON 0 h group (60.02% and 30.25%), the CON 48 h and ZSSF fermentation 48 h groups showed opposite trends for *Actinobacteria* (23.46%) and *Proteobacteria* (20.02%) (Figure S6B). It has been reported that certain *Firmicutes* can generate SCFAs by fermenting non-digestible carbohydrates in the gut, thus exerting a health-promoting effect on the host [40].

Figure 5C and Figure S6B show the changes in gut microbiota composition at the family and genus levels (top 20). In the CON group at 0 h, four gut microbiota types with high relative abundance were observed, namely, *Bifidobacterium* spp., *Shigella* spp., *Megasphaera* spp., and *Acidaminococcus* spp. Compared with the CON 0 h group, the relative abundances of *Megasphaera* spp. and *Acidaminococcus* spp. were significantly increased in both the CON 48 h and ZSSF fermentation 48 h groups, while the relative abundances of *Bifidobacterium* spp. and *Shigella* spp. were reduced (Figure 5C).

It has been reported that gut microbiota possesses remarkable capabilities, including the ability to facilitate glycosidic hydrolysis (*O*-glycosides and *C*-glycosides), ester cleavage, ring fission, and dehydroxylation of flavonoids [41]. It is plausible that *Bifidobacterium* spp., *Bacteroidaceae* (*Bacteroides* spp.), and *Enterobacteriaceae* may contribute to the hydrolysis of flavonoid *O*-glycosides in ZSSF, as these families have been reported to possess *O*-deglycosylation activity [42,43] and were among the top 20 gut microbial families in the ZSSF fermentation group (Figure S6B). Similarly, the gut microbiota also plays a crucial role in promoting the *O*-glycosidic hydrolysis of ZSSF in the present study. For instance, the spinosin (17) and its derivatives (9, 11, 13, 14, and 21), may be catalyzed by *Bifidobacterium* spp., *Bacteroidaceae* (*Bacteroides* spp.), and *Enterobacteriaceae*, as illustrated in Figure S6C.

The hydrolysis of *C*-glycosides is a prevalent metabolic reaction of flavonoids by gut microbiota [38]. In this study, the *C*-6 positions (vitexin (16) and swertisin (20)), *C*-8 positions (isovitexin (19) and isoswertisin (22)), and *C*-6,8 position (vicenin II (4)) are the major *C*-glycoside hydrolysis sites in ZSSF (Figure S2). The literature records also indicate that vitexin can be subjected to *C*-glycoside hydrolysis by *Lactobacillaceae* (*Lactobacillus* spp.) [44] and *Enterobacteriaceae* [45], resulting in the formation of apigenin. In addition, *Bacteroidaceae* (*Bacteroides* spp.) [7] and *Lachnospiraceae* (*Dorea* spp.) [46] possess the ability to hydrolyze flavonoid *C*-glycosides (Figure S6C), all of which were among the top families identified in the ZSSF fermentation group (Figure S6B). Therefore, it is plausible that *Lactobacillaceae* (*Lactobacillus* spp.), *Bacteroidaceae* (*Bacteroides* spp.), *Lachnospiraceae* (*Dorea* spp.), and *Enterobacteriaceae* potentially contributed the hydrolysis of *C*-glycosides in ZSSF (Figure S6C).

According to Figure S2, the spinosin derivatives mentioned above (23, 26, 30, 31, 32, 33, 34, 35, and 37) underwent ester cleavage to yield spinosin (17). Numerous microorganisms, such as *Lactobacillaceae* (*Lactobacillus* spp.), are capable of hydrolyzing chlorogenic acids via cinnamoyl esterase enzymes, and these enzymes act on the ester bond, resulting in the release of caffeic acid [47]. Meanwhile, *Lactobacillaceae* (*Lactobacillus* spp.) was also the predominant gut microbiota in the ZSSF fermentation group (Figure S6B). Thus, the production of spinosin might be attributed to the ester cleavage reaction catalyzed by *Lactobacillaceae* (*Lactobacillus* spp.) (Figure S6C).

Microbial metabolism eventually leads to the ring fission reaction of flavonoid aglycones such as quercetin (36), apigenin (38), kaempferol (39), and genkwanin (40) (Figure S2). According to the literature, *Enterobacteriaceae* can catalyze ring fission of quercetin, resulting in the production of phenylpropionic acid with two hydroxyl groups [45]. Furthermore, studies have also demonstrated the ability of *Bifidobacterium* spp. and *Bacteroidaceae* (*Bacteroides* spp.) to facilitate *C*-ring fission of flavonoids [48]. These bacteria exhibited high abundance at the family taxonomic level in the ZSSF fermentation group (Figure S6B). Based on these observations, we propose that *Bifidobacterium spp.*, *Bacteroidaceae* (*Bacteroides* spp.), and *Enterobacteriaceae* possess the ability to facilitate the ring fission reaction of flavonoid aglycones in ZSSF (Figure S6C).

The present study demonstrates that *p*-HPPA (M1) and *m*-HPPA (M2) undergo dehydroxylation and are converted to 3-PPA (M5) by gut microbiota (Figure S2). *Enterobacteriaceae* has been reported to possess the ability to perform dehydroxylation, which can convert naringin into dihydrokaempferol [49]. Therefore, it is hypothesized that the dehydroxylation of ZSSF by *Enterobacteriaceae* may serve as the underlying mechanism.

#### 3.3.5. Linear Discriminant Analysis

The present study performed linear discriminant analysis (LDA) to identify microbial taxa biomarkers. The results are shown in Figure 5D. A total of 28 gut microbiota with LDA scores above 4.0 showed significant differences among all groups. There were 12, 10, and 6 dominant statistically significant species in the CON 0 h (colored in red), CON 48 h (colored in green), and ZSSF fermentation 48 h (colored in blue) groups, respectively. The gut microbiota of the CON 0 h group included *Bifidobacterium* spp., *Shigella* spp., *Coriobacterium* spp., *Odoribacter* spp., *Enterobacteriaceae*, *Actinobacteria*, *Gammaproteobacteria*, and *Proteobacteriaceae* as the key differential taxa (Figure 5D). The CON 48 h group exhibited the most distinct microbiome composition. The predominant gut microbiota were mainly *Megasphaera* spp., *Acidaminococcus* spp., *Sphingobium* spp., *Dorea* spp., *Veillonellaceae*, *Lachnospiraceae*, *Clostridiales*, and *Firmicutes*.

*Ruminococcaceae* and *Verrucomicrobiaceae* (*Akkermansia* spp.) are the dominant types contributing to the differences in gut microbiota resulting from ZSSF 48 h intervention. Notably, although alpha diversity analysis revealed no significant differences in overall species richness or evenness between the CON and FZSSF groups at 48 h, the LDA results identified *Ruminococcaceae* and *Verrucomicrobiaceae* (*Akkermansia* spp.) as ZSSF-specific differential taxa. This indicates that ZSSF selectively modulated specific microbial populations even in the absence of global diversity changes, underscoring the importance of analyzing community composition beyond alpha diversity measures.

*Akkermansia* spp., belonging to the *Verrucomicrobiaceae* family, has been found to be involved in the metabolism of a variety of carbohydrates, with the ability to hydrolyze *O*-glycosidic bonds [50]. Moreover, *Akkermansia* spp. is a human gut microbiota that plays an important role as a beneficial microorganism in alleviating obesity and related metabolic disorders [51]. The efficacy of *Akkermansia* spp. as a modulator of gut homeostasis and a probiotic for human health has been demonstrated in several studies [52]. According to the literature, *Ruminococcaceae* has been identified as a microbial biomarker of chronic insomnia [53]. Moreover, *Ruminococcaceae* is a prominent producer of butyric acid [54] and propionic acid [55] in the gut microbiota, while also playing a crucial role in stabilizing the intestinal barrier through the breakdown of dietary cellulose within the host. The addition of ZSSF resulted in *Ruminococcaceae* being identified as an LDA differential gut microbiota in this study. Given its previous association with chronic insomnia [53], this finding suggests a hypothesis that ZSSF supplementation could potentially influence sleep quality through modulation of this taxon, though this remains to be proven in vivo.

### 3.4. Quantitative Analysis of Short-Chain Fatty Acids

**Figure 6:**
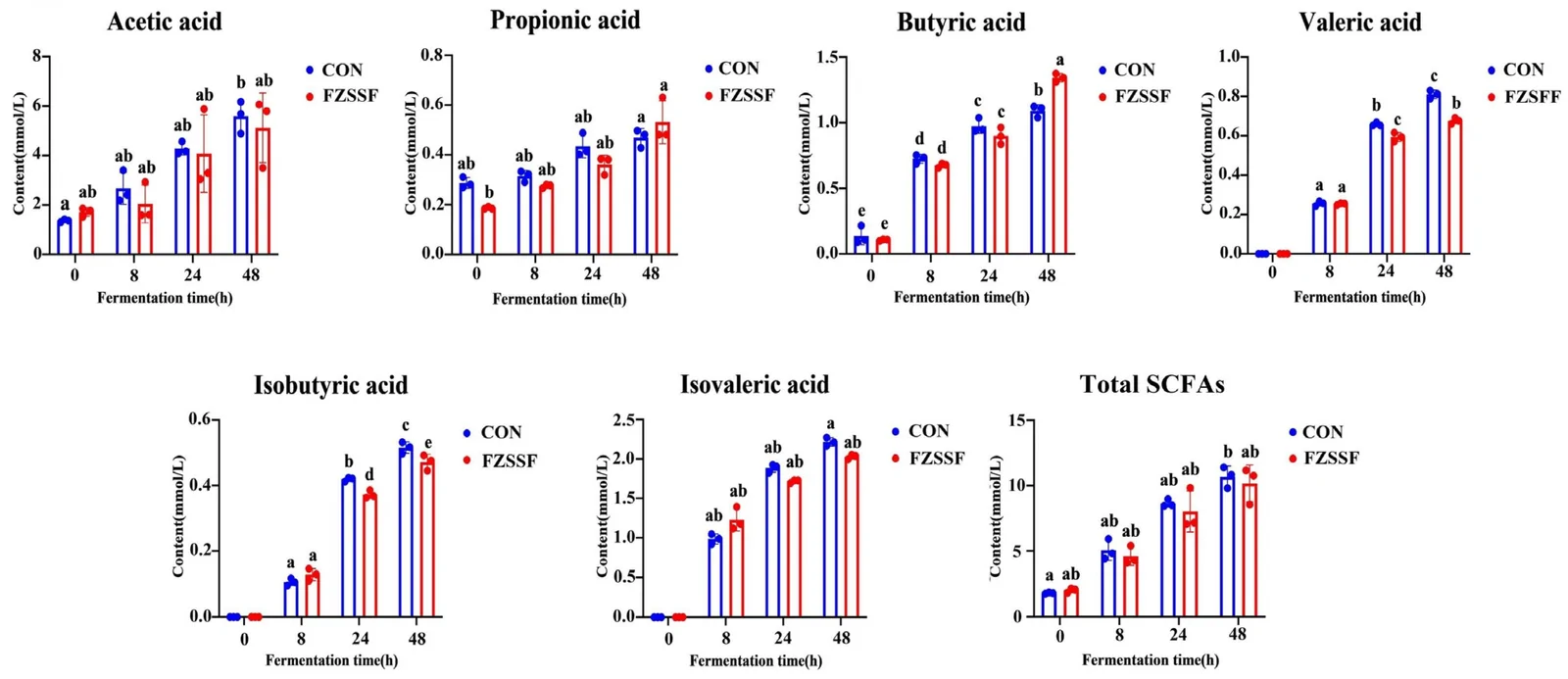
Concentrations of short-chain fatty acids (SCFAs) in the fermentation broth at different time points (different letters represent significant differences, *p* < 0.05).

Short-chain fatty acids (SCFAs), which are products of microbial activity, can enhance the physiological and metabolic functions of the host [56]. In this study, GC-MS was used to analyze the concentrations of total SCFAs and individual SCFAs (including acetic acid, propionic acid, butyric acid, valeric acid, isobutyric acid, and isovaleric acid) produced in the CON and ZSSF fermentation groups (Figure 6, Table S6).

The concentration of butyric acid in the fermented products of both groups showed an increasing trend. Butyric acid increased from 0.11 ± 0.01 mmol/L (0 h) to 1.34 ± 0.03 mmol/L in the ZSSF fermentation 48 h group, and was significantly higher than that in the CON 48 h group (1.10 ± 0.05 mmol/L, *p* < 0.05) (Table S6). The enhanced effect of ZSSF became more prominent as the fermentation time increased, based on the quantitative analysis results of butyric acid. It indicates that ZSSF specifically promoted butyric acid synthesis, which is consistent with the enrichment of butyric acid-producing taxa such as *Ruminococcaceae* and *Akkermansia* observed in the ZSSF fermentation group. Butyric acid is known to exert beneficial effects on intestinal health by serving as an energy source for colonocytes and maintaining gut barrier integrity [13].

However, after fermentation in the CON group, valeric acid and isobutyric acid increased from 0 mmol/L (0 h) to 0.81 ± 0.02 mmol/L and 0.70 ± 0.02 mmol/L (48 h), respectively, which were significantly higher than those in the ZSSF fermentation 48 h group (*p* < 0.05). After 48 h of fermentation, the concentrations of acetic acid, propionic acid, and isobutyric acid increased in the CON and ZSSF fermentation groups; however, there were no significant differences between them.

The enhanced butyric acid production in the ZSSF fermentation group suggests that ZSSF selectively promoted butyrate synthesis, which is consistent with the enrichment of butyrate-producing taxa such as *Ruminococcaceae* and *Akkermansia* observed in the ZSSF fermentation group. However, the correlations between specific microbial taxa and SCFAs production were not further analyzed in this study due to the exploratory nature of the analysis and the limited sample size.

It should be noted that the present findings are derived from an in vitro batch fermentation model. This system, while providing a controlled environment, is subject to limitations such as the absence of a host mucosal interface and the possibility of pH drift, gas build-up, and nutrient depletion over the 48 h incubation period. These factors, common in batch cultures, could non-specifically influence microbial community dynamics and SCFAs production. Furthermore, due to the limited sample size, no correlation analysis was performed between specific microbial taxa and SCFA production. Therefore, while our findings provide a mechanistic basis for the gut microbiota-mediated biotransformation of ZSSF, they require direct validation in in vivo studies. Future work should employ animal models to directly test the functional relevance of these in vitro observations.

## 4. Conclusions

In the present study, the relationship between ZSSF and the human gut microbiota was investigated using an in vitro anaerobic fermentation model. A total of 40 prototype components and five metabolites, including *p*-HPPA (**M1**), *m*-HPPA (**M2**), and 3-PPA (**M5**), were identified. The possible biotransformation pathways of ZSSF, involving both the flavonoid *C*-glycoside pathway and the flavonoid *O*-glycoside pathway, were inferred from the dynamic changes of various flavonoids in ZSS. The in vitro fermentation results showed that ZSSF was ultimately degraded to *m*-HPPA (**M2**) and 3-PPA (**M5**) by gut microbiota through ester cleavage, deglycosylation, demethylation, ring fission, and dehydroxylation reactions. This study revealed that genkwanin and apigenin derivatives with a 6-*C*-glycoside structure were metabolized faster by gut microbiota than those with an 8-*C*-glycoside structure. In addition, ZSSF and their catabolic metabolites regulated gut microbiota composition by increasing the relative abundance of *Firmicutes* while decreasing the relative abundances of *Actinobacteria* and *Proteobacteria*. LDA analysis indicated that *Ruminococcaceae* and *Verrucomicrobiaceae* (*Akkermansia* spp.) were the dominant families in the presence of ZSSF after incubation. Moreover, it was observed that ZSSF was associated with higher butyrate concentration at 48 h under the tested batch fermentation conditions. Notably, *Ruminococcaceae* and *Verrucomicrobiaceae* (*Akkermansia* spp.) exhibited a strong positive correlation with butyric acid. These results suggest that ZSSF and their catabolic metabolites may contribute to health benefits due to their ability to positively regulate gut microbiota composition and metabolites (SCFAs). It should be noted that the present findings are derived from an in vitro fermentation model. Therefore, in vivo animal studies, particularly using insomnia or anxiety models, are required to directly validate the sleep-improving effects of ZSSF and to establish the causal relationship between gut microbiota modulation and host behavioral changes. Collectively, this study provides a mechanistic basis for the gut microbiota-mediated biotransformation and activity of ZSSF, offering a reference for further in vivo studies.

## Data Availability

The original contributions presented in this study are included in the article/Supplementary Materials, and further inquiries can be directed to the corresponding authors.

## Supplementary Materials

The following supporting information can be downloaded at: https://www.mdpi.com/article/10.3390/foods15173077/s1, Figure S1: Chemical structures of ZSSF and their metabolites; Figure S2: Proposed biotransformation pathways of flavonoid-C-glycosides and flavonoid-O-glycosides from ZSSF during *in vitro* fermentation (Compounds with commercial standards are shown in blue, and others are shown in black. Solid arrows represent confirmed pathways, and dashed arrows represent speculated pathways.); Figure S3: Degradation curves of (A) spinosin derivatives, (B) isospinosin and isoswertisin, (C) isovitexin derivatives, and (D) kaempferol derivatives in ZSSF (n = 3); Figure S4: Effect of fermentation on gut microbiota. (A) Rarefaction curves of samples; (B) Shannon curves of samples; (C) Alpha diversity indices; (D) Venn diagram; Figure S5: (A) Non-metric multidimensional scaling (NMDS); (B) Hierarchical clustering trees; Figure S6: (A) Relative abundance at the phylum level; (B) Relative abundance of the gut microbiota community at the family level; (C) Relative abundance of gut microbiota that hydrolyze ZSSF. Asterisks indicate significantly (* *p* < 0.05) or highly significantly (** *p* < 0.01) different relative abundances; Table S1: Characterization of the chemical constituents of ZSSF and metabolites fermented by human gut microbiota (HGM) based on UPLC-Q-TOF-MS/MS; Table S2: Calibration curves for 10 flavonoids and 3 metabolites in ZSSF; Table S3: Precision for the determination of 10 flavonoids and 3 metabolites in ZSSF; Table S4: Concentrations of 10 flavonoids and 3 metabolites in ZSSF after incubation with human gut microbiota at different time points (standards were used for reference; n = 3); Table S5: Relative concentrations of ZSSF metabolites after incubation with human gut microbiota at different time points (no reference standards were available; data are presented as relative abundances; n = 3); Table S6: Concentrations of SCFAs in the fermentation broth at different time points (n = 3).

## Abbreviations

The following abbreviations are used in this manuscript

ZSS, Ziziphi spinosae semen; ZSSF, Ziziphi spinosae semen flavonoids; UPLC-Q-TOF-MS/MS, ultra-high performance liquid chromatography coupled with quadrupole time-of-flight mass spectrometry; SCFAs, short-chain fatty acids; 3-PPA, 3-phenylpropionic acid; *m*-HPPA, *m*-hydroxyphenylpropionic acid; *p*-HPPA, *p*-hydroxyphenylpropionic acid; CON, the group none of ZSSF solution included; LDA, linear discriminant analysis; GABA, γ-aminobutyric acid; RSD, relative standard deviation.
