# Supplementary material for: Metabolic Profile and Dynamic Characteristics of Ziziphi Spinosae Semen Flavonoids During Biotransformation and Their Effect on Human Gut Microbiota In Vitro
Source: Foods. 2026 Aug 30;15(17):3077. doi: 10.3390/foods15173077 (PMC13564612; doi:10.3390/foods15173077)
Supplement: Supplementary file 1 [file foods-15-03077-s001.zip › foods-4434647-supplementary.pdf]

Supplementary Materials

Metabolic Profile and Dynamic Characteristics of Ziziphi Spinosae Semen Flavonoids During Biotransformation and Their Effect on Human Gut Microbiota In Vitro

Ruiqi Yang [1,†], Haolin Zhang [1,†], Yadan Mo [1], Conghui Wang [1], Jinchun Wu [1], Lijun Guo [1], Xiangping Pei [1], Yan Yan[2,*] and Chenhui Du [1,*]

Table S1. Characterization of the chemical constituents of ZSSF and metabolites fermented by human gut microbiota (HGM) based on UPLC-Q-TOF-MS/MS.

No.	Compounds	t_R (min)	$[M-H]^-$ (*m*/*z*)	Formula	Error (ppm)	MS/MS fragment ions (*m*/*z*)
1	Gallic acid	3.35	169.0143	$C_7H_6O_5$	0.4	**125.0222**
2	Protocatechuic acid	5.51	153.0200	$C_7H_6O_4$	4.3	**108.0216,91.0239**
3	Catechin	8.136	289.0713	$C_{15}H_{14}O_6$	-1.6	245.0807,**203.0695,125.0213**
4	Vicenin II*	10.11	593.1505	$C_{27}H_{30}O_{15}$	-1.1	**473.1084,383.0752,353.0665,503.1171,297.0780**
5	Hovetrichoside C	10.17	449.1079	$C_{21}H_{22}O_{11}$	-2.4	**287.0557,269.0456,259.0590**,178.9991,125.0242
6	*p*-coumarin acid	12.10	163.0403	$C_9H_8O_3$	1.5	**119.0507,93.0325**
7	Ethyl gallate	12.20	197.0455	$C_9H_{10}O_5$	-0.4	169.0122,**125.0246**,124.0153,78.0125
8	Vanillic acid	12.37	167.0353	$C_8H_8O_4$	1.8	167.0342,152.0135,**108.0218**
9	6'''-(4''''-*O*-*β*-*D*-glucopyranosyl)-vanilloylspinosin	12.97	921.2506	$C_{42}H_{48}O_{23}$	-0.9	757.2011,**427.1000,307.0628,292.0335**,209.0447,167.0330
10	Isovitexin-2''-*O*-*β*-*D*-glucopyranoside	13.41	593.1507	$C_{27}H_{30}O_{15}$	-0.8	473.1084,413.0876,**311.0544**,293.0451,282.0518
11	6'''-(4''''-(*O*-glc)-*p*-	13.46	889.241	$C_{41}H_{46}O_{22}$	0.7	769.1706,**727.1925**,607.1523,**427.1061**,325.0508,**307.0563,292.0621,239.0533**,209.06

	hydroxybenzoylspinosin		4			56
12	Camelliaside B	13.93	725.1938	$C_{32}H_{38}O_{19}$	9.5	**575.1426**,284.0323,**255.0288**,227.0346
13	6'''-(4''''-(*O*-glc)-feruloylspinosin	13.94	945.2672	$C_{44}H_{50}O_{23}$	0.2	783.2180,663.1816,607.1808,487.1318,**427.0990,307.0514,292.0215,**235.0726
14	6''-(4''''-(*O*-glc)-*p*-coumaloylspinosin	13.99	915.2562	$C_{43}H_{48}O_{22}$	-0.2	753.2039,725.2113,**607.1719,**427.0956
15	Isospinosin	14.18	607.1660	$C_{28}H_{32}O_{15}$	-1.5	487.1254,**427.1049**,324.0643,**307.0608,292.0443**
16	Vitexin*	14.30	431.098	$C_{21}H_{20}O_{10}$	-0.9	**413.0983,341.0653,311.0325,283.0659**
17	Spinosin*	14.32	607.1686	$C_{28}H_{32}O_{15}$	-1.1	487.1279,**427.1042**,307.0617,**307.0617,292.0376**,325.0715
18	Rutin*	14.36	609.1498	$C_{27}H_{30}O_{16}$	6.1	489.1275,**429.1076**,326.0700,**301.0338,300.0266**,294.0418,282.0505, 271.0240,179.9965,151.0023
19	Isovitexin*	14.57	431.0974	$C_{21}H_{20}O_{10}$	-2.7	413.0891,**341.0653**,**311.0553**,**283.0608**
20	Swertisin*	15.25	445.1135	$C_{22}H_{22}O_{10}$	-1.2	**325.0704**,**297.0397**,**282.0528**,**269.0447**,231.0268
21	6'''-(4''''-*O*-rha)-vanilloylspinosin	15.53	903.2579	$C_{42}H_{48}O_{22}$	1.6	757.2079,**427.1138**
22	Isoswertisin	16.01	445.1127	$C_{22}H_{22}O_{10}$	-3.1	**325.0714**,**297.0366**,**282.0521**
23	6'''-vanilloylspinosin	16.45	757.1993	C36H38O18	1.0	637.1575,607.1730,589.1592,**427.1027**,325.0696,307.0605,292.0351,**269.0661**, 255.0511,239.0556,**209.0437**,181.0508,167.0333
24	Kaempferol-3-*O*-rutinoside*	16.56	593.1542	$C_{27}H_{30}O_{15}$	-0.2	**285.0397**,255.0291,229.0490,327.0497

25	Isoscoparin-2''-*O*-(6-feruloyl)-glucopyranoside	16.85	799.2125	$C_{38}H_{40}O_{19}$	0.6	**679.1673**,**593.1527**,**413.0894**,325.0878,265.0684,223.0581
26	6'''-p-hydroxybenzoylspinosin	16.94	727.1878	$C_{35}H_{36}O_{17}$	-0.3	**427.1033**,**307.0602,292.0366,**239.0559,179.0349
27	Isovitexin-2''-*O*-(6-*p*-coumaloyl)-glucopyranoside	17.19	739.1882	$C_{36}H_{36}O_{17}$	0.3	**593.1483**,473.1239,**413.0862**,**341.0658,311.0256,293.0465,**205.0498
28	Isovitexin-2''-*O*-(6-feruloyl)-glucopyranoside	17.38	769.198	$C_{37}H_{38}O_{18}$	-0.7	**593.1508,413.0864,341.0643,**323.0658,**311.0256**
29	Naringin	17.61	579.1712	$C_{27}H_{32}O_{14}$	-1.3	**459.1008**,415.1067,**271.0609**,177.0216,151.0030
30	6'''-sinapoylspinosin	18.53	813.225	$C_{39}H_{42}O_{19}$	0.3	607.1657,**427.1019,**325.0868,**307.0771,292.0354,**265.0697,190.0330
31	6'''-dihydrophaseoylspinosin	18.57	871.3037	$C_{43}H_{52}O_{19}$	0.8	607.1689,**427.1028,**325.0705,**307.0372,292.0425**
32	6'''-*p*-coumaloylspinosin	18.94	753.2034	$C_{37}H_{38}O_{17}$	-0.3	607.1679,**427.1031,307.0813,292.0363,**265.0698,205.0496,163.0391
33	6'''-feruloylspinosin*	19.16	783.2146	$C_{38}H_{40}O_{18}$	0.5	607.1719,**427.1040**,**307.0608,**295.0822,**292.0380**,235.0614,193.0512,161.0247
34	6'''-(–)-phaseoylspinosin	21.34	869.2876	$C_{43}H_{50}O_{19}$	0.3	607.1605,589.1480,**427.0987,307.0539,292.0384,**279.1266
35	6'''-(3'''',4'''',5''''-trimethoxyl)-cinnamoylspinosin	22.27	827.2412	$C_{40}H_{44}O_{19}$	0.9	707.2336,445.1212,**427.0990**,**307.0523,**295.0832,**292.0830**,281.0532,265.0712

36	Quercetin*	23.33	301.0344	$C_{15}H_{10}O_7$	-3.1	255.2249,**216.9895**,**178.0034**,164.9524,**151.0041**
37	6'''-cinnamoylspinosin	23.34	737.2092	$C_{37}H_{38}O_{16}$	0.7	617.1978,589.1517,487.8485,**427.0945**,367.1909,**307.0609**
38	Apigenin*	24.61	269.0461	$C_{15}H_{10}O_5$	2	**228.9940**,182.9828,**151.0039**,**117.0345**
39	Kaempferol*	24.68	285.0398	$C_{15}H_{10}O_6$	-2.5	**244.9822**,**223.0297**,**216.9903**,178.9913,132.9842,117.0348
40	Genkwanin*	25.54	283.061	$C_{16}H_{12}O_5$	-0.6	**268.0381**,**239.0321**,**215.1638**,178.9905,151.0022,116.9995
M1	*p*-hydroxyphenylpropionic acid*	11.169	165.0536	$C_9H_{10}O_3$	2.5	121.0674,**96.9709**,**93.0330**,**78.9597**,59.0140
M2	*m*-hydroxyphenylpropionic acid*	13.062	165.0533	$C_9H_{10}O_3$	1.7	**121.0653**,119.0487,**106.0423**,96.9673,78.9597,65.0446
M3	6'''-feruloylspinosin-CH_2O	18.92	753.2032	$C_{37}H_{38}O_{17}$	-0.50	633.1674,607.1708,589.1584,427.1021,325.0747,307.0592,265.0702,163.0393
M4	Swertisin+4H	19.64	449.1450	$C_{22}H_{26}O_{10}$	-0.70	359.1125,329.1022,235.0608,223.0602,191.0331,180.0425
M5	3-phenylpropionic acid*	22.519	149.0572	$C_9H_{10}O_2$	3.2	**89.0288**

* Confirmed by comparison of their retention times and MS data with commercial standards. Bold indicates characteristic ions. RT: retention time; Rha: Rhamnoside; Rut: rutinoside; Glu: glucoside; M: metabolites.

Table S2. Calibration curves for 10 flavonoids and 3 metabolites in ZSSF.

No.	Analyte	Regression equation	*r*	Linearange (ng/mL)
4	Vicenin II	Y=404.57957X-280.96107	0.9999	3.0~404.8
16	Vitexin	Y=631.28628X-75.49257	0.9994	1.0~95.0
17	Spinosin	Y=467.91560X+12.64680	0.9994	29.0~7744.0
19	Isovitexin	Y=1110.92323X-403.91648	0.9998	1.0~306.6
20	Swertisin	Y=675.05203X+396.01849	0.9992	1.0~3968.0
24	Kaempferol-3-*O*-rutinoside	Y=315.64164X-275.99376	0.9999	3.0~5030.0
33	6'''-feruloylspinosin	Y=414.19906X-1212.09917	0.9996	22.4~5600.0
38	Apigenin	Y=2547.24835X+8178.96885	0.9990	10.0~803.2
39	Kaempferol	Y=1968.35403X+2032.66711	0.9994	9.9~788.8
40	Genkwanin	Y=2574.55847X+1243.54589	0.9990	10.0~796.8
M1	*p*-HPPA	Y=105.22043X-139.28376	0.9992	10.3~1026.0
M2	*m*-HPPA	Y=392.33277X-364.90869	0.9997	10.9~1092.0
M5	3-PPA	Y=45.06172X-1271.05787	0.9990	60.8~3042.0

Table S3. Precision for the determination of 10 flavonoids and 3 metabolites in ZSSF.

No.	Analyte	Concentration (ng/mL)	Inter-day Precision (%)	Intra-day Precision (%)
4	Vicenin II	151.8	2.91	2.35
16	Vitexin	9.5	5.70	6.74
17	Spinosin	2904.0	1.43	2.45
19	Isovitexin	20.4	0.47	1.98
20	Swertisin	297.6	1.51	2.93
24	Kaempferol-3-*O*-rutinoside	301.8	3.21	3.25
33	6'''-feruloylspinosin	2240.0	3.58	3.39
38	Apigenin	502.0	2.21	3.00
39	Kaempferol	493.0	3.75	4.41
40	Genkwanin	498.0	2.84	4.79
M1	*p*-HPPA	513.0	1.90	1.50
M2	*m*-HPPA	546.0	2.34	2.35
M5	3-PPA	1521.0	1.35	1.43

Table S4. Concentrations of 10 flavonoids and 3 metabolites in ZSSF after incubation with human gut microbiota at different time points (standards were used for reference; n = 3).

No.	Analyte	Concentration (μg/mL)						
		0 h	2 h	4 h	8 h	12 h	24 h	48 h
4	Vicenin II	2.32±0.09	2.25±0.09	2.25±0.03	2.49±0.09	2.38±0.03	1.33±0.31	<LOD
16	Vitexin	0.10±0.01	0.57±0.01	1.02±0.01	1.05±0.00	1.05±0.02	0.96±0.17	<LOD
17	Spinosin	61.38±1.67	58.71±1.74	59.01±0.65	51.63±2.21	42.15±16.48	0.72±0.16	<LOQ
19	Isovitexin	0.39±0.01	0.52±0.00	0.77±0.03	1.45±0.08	2.7±0.21	<LOD	<LOD
20	Swertisin	0.93±0.01	1.21±0.13	5.27±0.38	12.96±1.87	43.37±5.47	55.00±3.55	0.27±0.01
24	Kaempferol-3-*O*-rutinoside	3.44±0.17	<LOD	<LOD	<LOD	<LOD	<LOD	<LOD
33	6'''-feruloylspinosin	22.00±0.43	22.55±0.18	21.93±0.09	18.85±0.86	17.38±0.26	3.08±1.07	<LOD
38	Apigenin	<LOQ	<LOD	<LOD	<LOD	<LOD	<LOD	<LOD
39	Kaempferol	0.25±0.02	<LOD	<LOD	<LOD	<LOD	<LOD	<LOD
40	Genkwanin	<LOD	<LOD	<LOD	<LOD	<LOQ	<LOD	<LOD
M1	*p*-HPPA	<LOD	<LOD	<LOD	1.73±0.07	2.07±0.11	8.19±1.43	0.3±0.10
M2	*m*-HPPA	<LOD	<LOD	<LOD	0.45±0.03	0.66±0.03	3.67±0.17	5.02±0.70
M5	3-PPA	2.67±0.19	2.72±0.25	4.55±0.25	7.59±0.36	8.89±0.72	12.37±1.62	49.32±0.54

Table S5. Relative concentrations of ZSSF metabolites after incubation with human gut microbiota at different time points (no reference standards were available; data are presented as relative abundances; n = 3).

No.	Analyte	Concentration (μg/mL)						
		0 h	2 h	4 h	8 h	12 h	24 h	48 h
10	Isovitexin-2''-*O*-*β*-*D*-glucopyranoside	2.23±0.06	2.26±0.15	2.05±0.15	2.02±0.18	0.43±0.12	0.04±0.01	<LOD
12	Camelliaside B	2.70±0.08	2.19±0.25	1.36±0.30	0.60±0.16	0.29±0.18	0.05±0.05	<LOD
15	Isospinosin	7.70±0.50	4.34±0.74	<LOD	<LOD	<LOD	<LOD	<LOD
22	Isoswertisin	0.36±0.07	3.94±0.43	8.39±0.35	9.51±0.30	9.31±0.32	9.59±0.73	0.03±0.01
23	6'''-Vanilloylspinosin	1.31±0.08	1.78±0.09	1.83±0.15	2.00±0.07	1.81±0.09	1.21±0.44	<LOD
27	Isovitexin-2''-*O*-(6-*p*-coumaloyl)-glucopyranoside	0.09±0.01	0.08±0.01	0.08±0.01	0.08±0.01	0.07±0.01	0.03±0.02	<LOD
28	Isovitexin-2''-*O*-(6-feruloyl)-glucopyranoside	0.48±0.02	0.46±0.02	0.45±0.03	0.45±0.00	0.39±0.01	0.18±0.10	<LOD
30	6'''-Sinapoylspinosin	1.05±0.06	1.03±0.02	1.00±0.04	0.98±0.02	0.85±0.02	0.35±0.02	<LOD
31	6'''-dihydrophaseoylspinosin	1.09±0.06	1.04±0.02	1.05±0.04	1.15±0.03	1.09±0.05	1.12±0.01	1.08±0.04
32	6'''-*p*-Coumaloylspinosin	5.03±0.21	4.92±0.40	4.56±0.36	4.23±0.18	3.22±0.15	0.71±0.51	<LOD

Table S6. Concentrations of SCFAs in the fermentation broth at different time points (n = 3).

Treatment	Time (h)	SCFAs (mmol/L)						
		Acetic acid	Propionic acid	Butyric acid	Valeric acid	Isobutyric acid	Isovaleric acid	Total SCFAs
CON	0	1.37±0.02^{a}	0.29±0.02^{ab}	0.14±0.07^{e}	ND	ND	ND	1.80±0.11^{a}
	8	2.67±0.16^{ab}	0.32±0.02^{ab}	0.73±0.03^{d}	0.26±0.01^{a}	0.11±0.01^{a}	0.99±0.06^{ab}	5.08±0.30^{ab}
	24	4.28±0.06^{ab}	0.44±0.05^{ab}	0.97±0.06^{c}	0.66±0.01^{b}	0.42±0.01^{b}	1.89±0.05^{ab}	8.66±0.24^{ab}
	48	5.58±0.50^{b}	0.47±0.04^{a}	1.10±0.05^{b}	0.81±0.02^{c}	0.52±0.02^{c}	2.22±0.05^{a}	10.70±0.68^{b}
FZSSF	0	1.72±0.18^{ab}	0.19±0.01^{b}	0.11±0.01^{e}	ND	ND	ND	2.02±0.20^{ab}
	8	2.04±0.77^{ab}	0.28±0.01^{ab}	0.67±0.02^{d}	0.25±0.01^{a}	0.13±0.02^{a}	1.23±0.14^{ab}	4.60±0.97^{ab}
	24	4.08±1.57^{ab}	0.36±0.04^{ab}	0.90±0.06^{c}	0.59±0.02^{c}	0.37±0.01^{d}	1.72±0.02^{ab}	8.02±1.72^{ab}
	48	5.12±1.41^{ab}	0.53±0.09^{a}	1.34±0.03^{a}	0.70±0.02^{b}	0.47±0.02^{e}	2.03±0.03^{ab}	10.19±1.60^{ab}

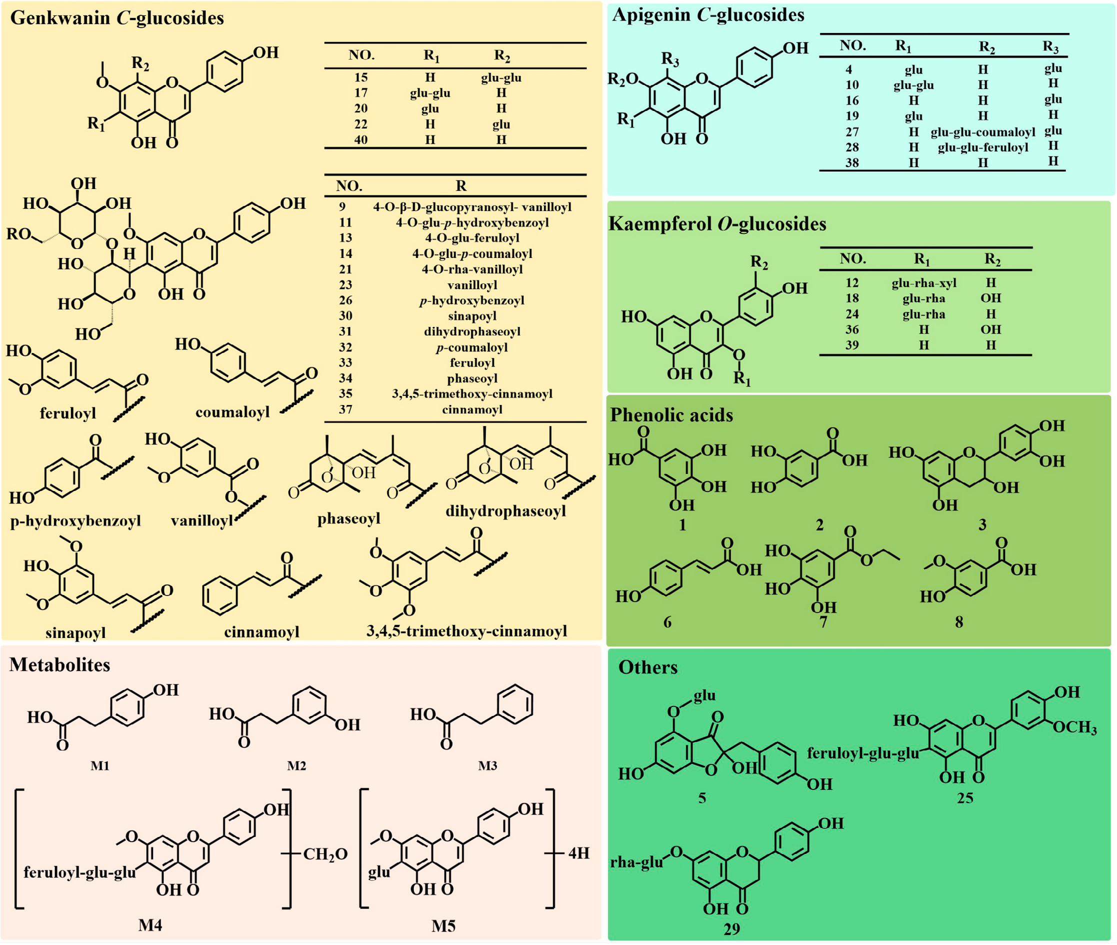

Figure S1. Chemical structures of ZSSF and their metabolites.

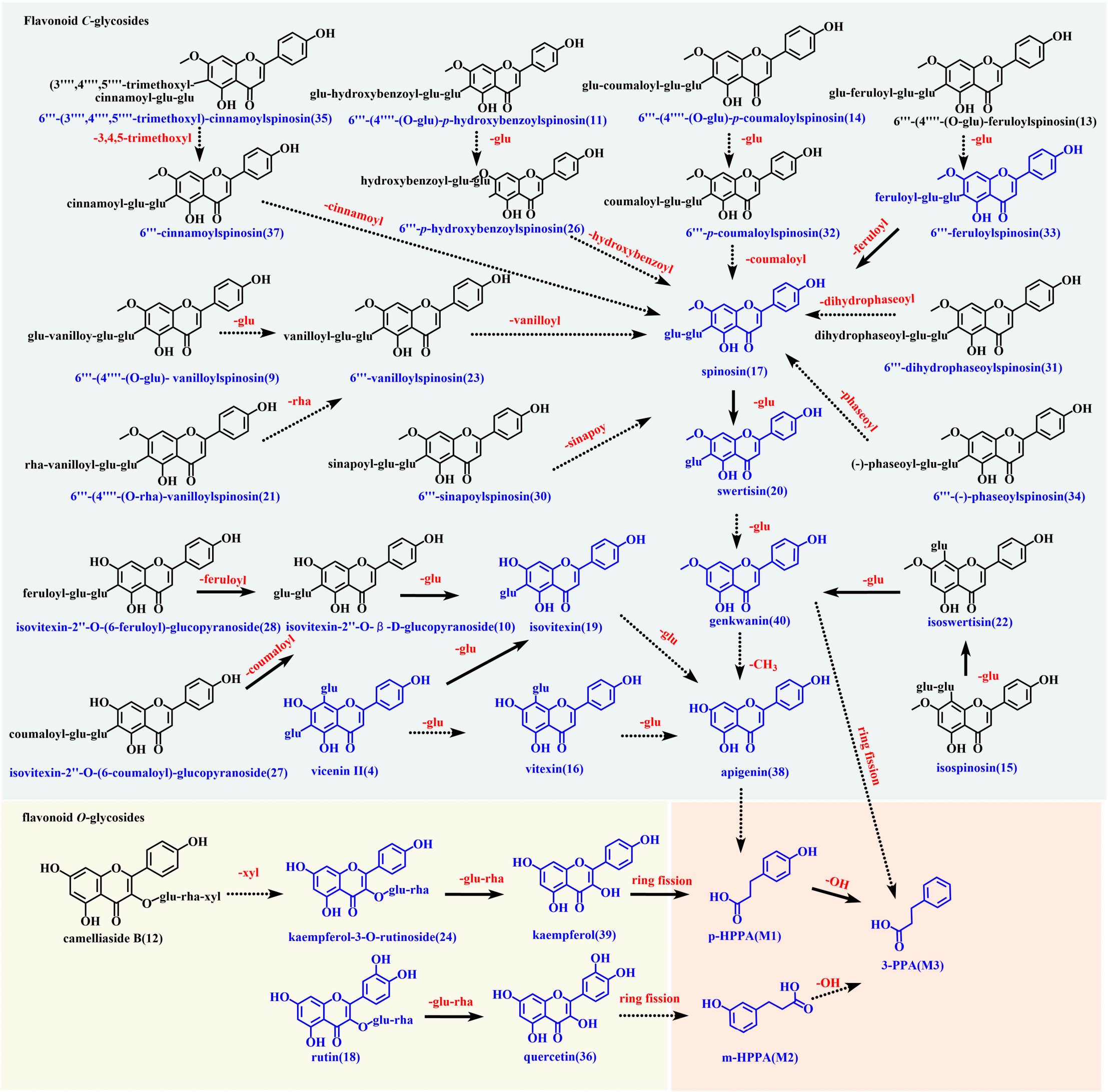

Figure S2. Proposed biotransformation pathways of flavonoid-*C*-glycosides and flavonoid-*O*-glycosides from ZSSF during *in vitro* fermentation. (Compounds with commercial standards are shown in blue, and others are shown in black. Solid arrows represent confirmed pathways, and dashed arrows represent speculated pathways.)

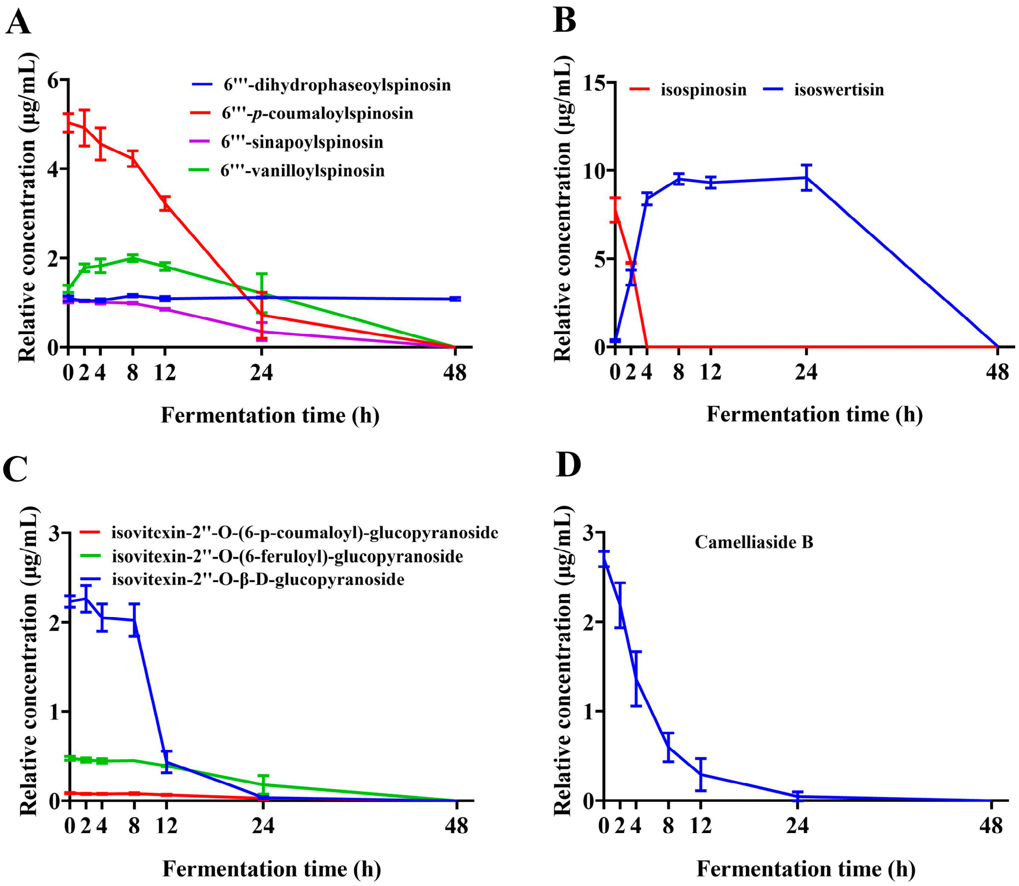

Figure S3. Degradation curves of (A) spinosin derivatives, (B) isospinosin and isoswertisin, (C) isovitexin derivatives, and (D) kaempferol derivatives in ZSSF (n = 3).

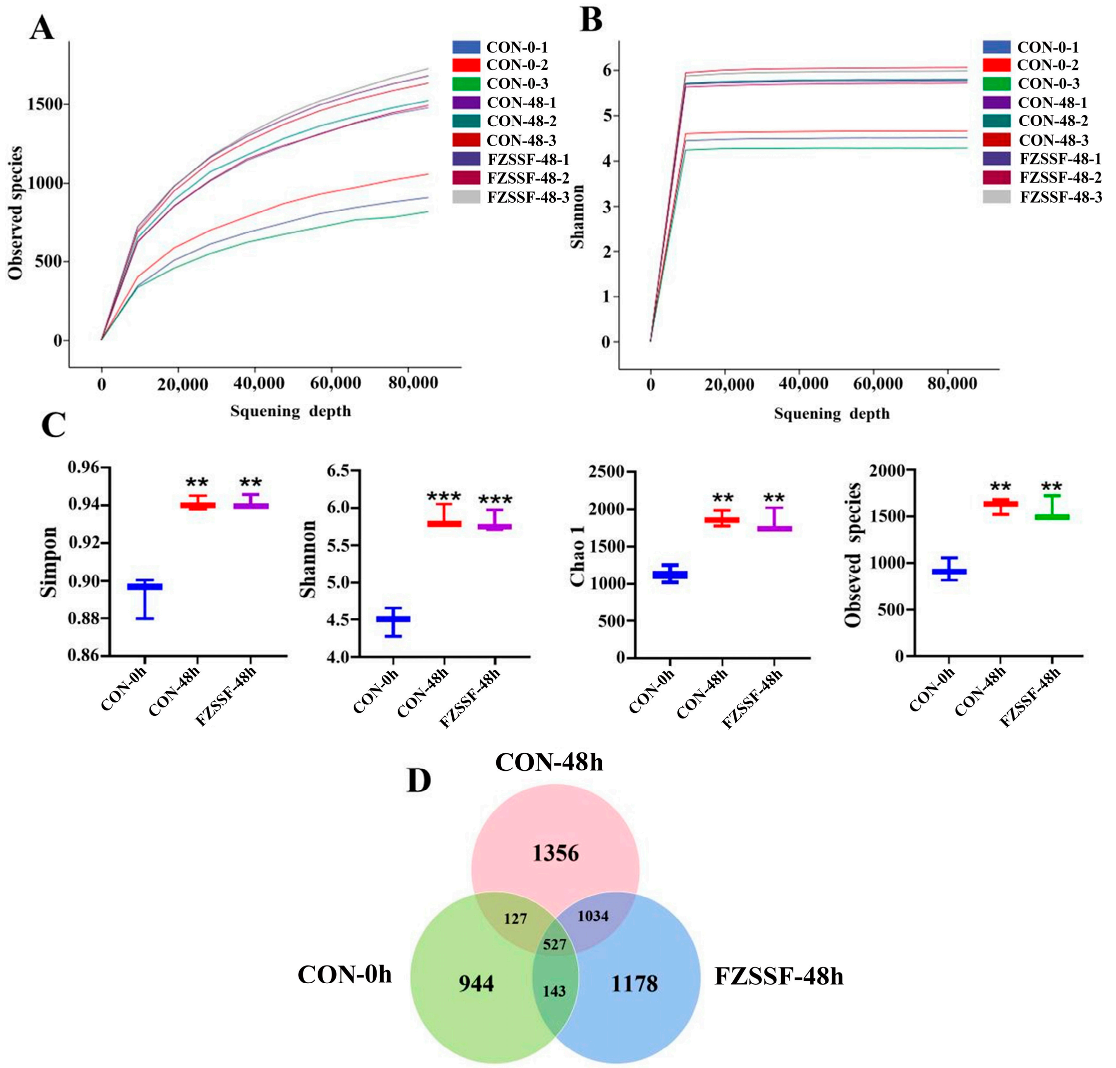

Figure S4. Effect of fermentation on gut microbiota. (A) Rarefaction curves of samples; (B) Shannon curves of samples; (C) Alpha diversity indices; (D) Venn diagram.

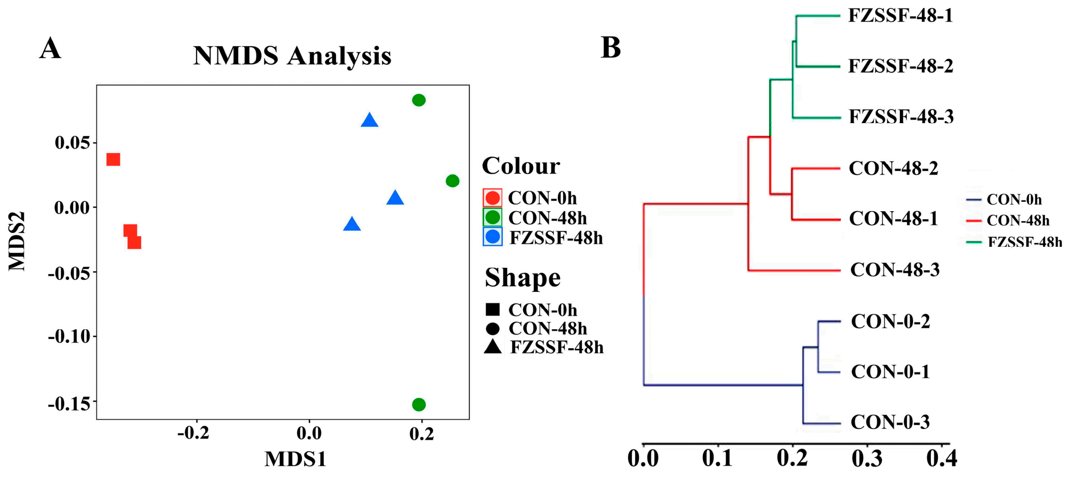

Figure S5. (A) Non-metric multidimensional scaling (NMDS); (B) Hierarchical clustering trees.

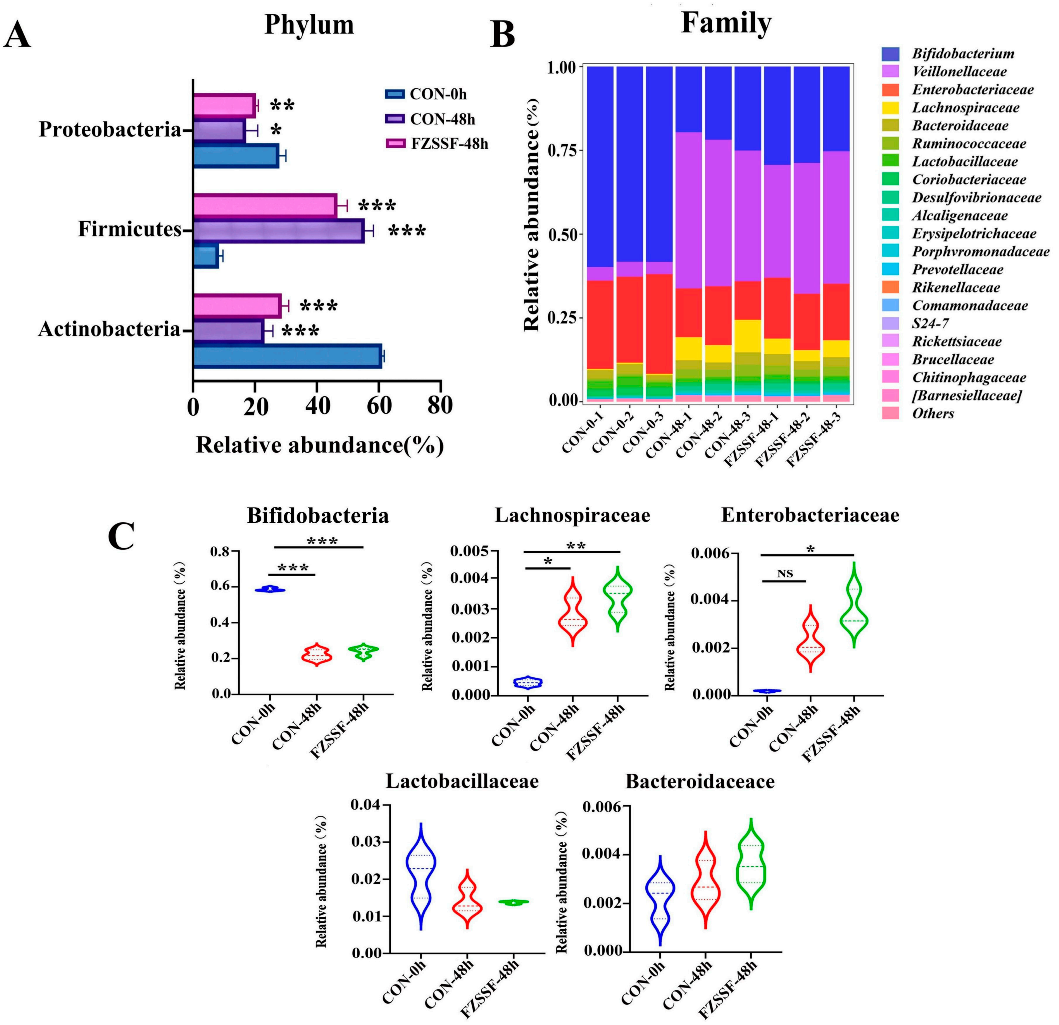

Figure S6. (A) Relative abundance at the phylum level; (B) Relative abundance of the gut microbiota community at the family level; (C) Relative abundance of gut microbiota that hydrolyze ZSSF. Asterisks indicate significantly (*$P < 0.05$) or highly significantly (**$P < 0.01$) different relative abundances.
